# Reproductive intentions and corresponding use of safer conception methods and contraception among Ugandan HIV clients in serodiscordant relationships

**DOI:** 10.1186/s12889-021-10163-7

**Published:** 2021-01-19

**Authors:** Glenn J. Wagner, Deborah Mindry, Emily A. Hurley, Jolly Beyeza-Kashesya, Violet Gwokyalya, Sarah Finocchario-Kessler, Rhoda K. Wanyenze, Mastula Nanfuka, Mahlet G. Tebeka, Kathy Goggin

**Affiliations:** 1 RAND Corporation, 1776 Main St, Santa Monica, CA 90407 USA; 2 UC Global Health Institute, Center for Women’s Health Gender and Empowerment, Los Angeles, CA USA; 3 Children’s Mercy Research Institute, Children’s Mercy Kansas City, Kansas City, USA; 4 Mulago Hospital Department of Obstetrics and Gynaecology, Makerere University College of Health Sciences, Kampala, Uganda; 5 Department of Disease Control and Environmental Health, Makerere University School of Public Health, Kampala, Uganda; 6 Department of Family Medicine, University of Kansas Medical Center, Kansas City, USA; 7 The AIDS Support Organization, Kampala, Uganda; 8 Schools of Medicine and Pharmacy, University of Missouri – Kansas City, Kansas City, USA

**Keywords:** Contraception, Serodiscordant, HIV, Safer conception methods, Family planning

## Abstract

**Context:**

Among people living with HIV in Uganda, desires to have a child and unplanned pregnancies are both common, while utilization of safer conception methods (SCM) and modern contraceptives are low.

**Methods:**

Three hundred eighty-nine HIV clients who reported considering childbearing with their uninfected partner enrolled in a safer conception counseling intervention trial in Uganda. Multiple regression analysis and baseline data were used to examine correlates of reproductive intentions and behaviors, including use of safer conception methods and contraception.

**Results:**

Most (*n* = 313; 80.5%) reported that both they and their partner wanted to have a child now, which was associated with being married, in a longer relationship, not having a child with partner, greater SCM knowledge, lower internalized childbearing stigma, and higher perceived community stigma of childbearing. However, just 117 reported trying to conceive in the prior 6 months, which was associated with being female, not having a child with their partner, less decision-making control within the relationship, and greater perceived cultural acceptability of SCM. Among those who had tried to conceive in the past 6 months, 14 (11.9%) used SCM, which was associated with greater control in decision making. Of the 268 who were not trying to conceive, 69 (25.7%) were using a modern contraceptive, which was associated with being in a shorter relationship, less control over decision-making, more positive attitudes towards contraception and lower depression.

**Conclusion:**

Methods to promote reproductive goals are underused by HIV serodiscordant couples, and relationships characteristics and childbearing-related stigma appear to be most influential and thus targets for intervention.

**Trial registration:**

Clinicaltrials.gov, NCT03167879; date registered May 23, 2017.

**Supplementary Information:**

The online version contains supplementary material available at 10.1186/s12889-021-10163-7.

## Plain English summary

Among people living with HIV in Uganda, unplanned pregnancies and desires to have a child and are both common. However, use of modern contraceptives and methods to safely conceive [safer conception methods (SCM)] are low. A sample of 389 HIV clients who reported considering childbearing with their uninfected partner was enrolled. Data were examined to identify factors associated with whether or not the client and their partner wanted to and were actively engaged in trying to conceive a child, and their corresponding use of SCM or contraception.

Most (*n* = 313) reported that both they and their partner wanted to have a child now, which was associated with being married, in a longer relationship, not having a child with partner, greater knowledge of SCM and less perceived stigma of childbearing. Less than a third (*n* = 117 of 385 with data; 30.4%) participants reported trying to conceive in the prior 6 months, which was associated with being female, not having a child with their partner, less decision-making control within their relationship, and greater perceived acceptance of SCM in their community.

Among those who had tried to conceive, only 14 (11.9%) used SCM, which was associated with greater control in decision making within their relationship. Of the 268 who were not trying to conceive, 69 (25.7%) were using a modern contraceptive, which was associated with being in a shorter relationship, less control over decision-making, more positive attitudes towards contraception and lower depression.

In conclusion, methods to promote reproductive goals are underused by couples living with HIV, and relationship characteristics and childbearing-related stigma appear to be most influential and thus targets for intervention.

## Introduction

Across sub-Saharan Africa, including Uganda, reproductive health and family planning services have been integrated into HIV care [1]. In Uganda, and throughout the region, family planning services historically and currently focus exclusively on preventing unplanned pregnancies. Services to support safer conception are not present, despite 20–60% of people living with HIV (PLHIV) in Uganda wishing to conceive [2–7] and up to 40% of HIV-positive women becoming pregnant post-HIV diagnosis [7, 8]. About half of these pregnancies are planned [7], highlighting the need for family planning programs to include safer conception counseling (SCC), while the unplanned pregnancies reveal a high unmet need for more effective contraception promotion. With 60% of HIV-affected couples in Uganda being serodiscordant [9], there is a clear need for a more expansive view of family planning services that help PLHIV and their partners to make informed childbearing decisions, and use effective methods for either safely conceiving and delivering a child, or preventing unwanted pregnancies.

Antiretroviral therapy (ART) greatly reduces the transmission risks related to childbearing [10], but in Uganda ~ 30% of PLHIV are not on ART [11], ~ 30% of those on ART have suboptimal adherence (including women on ART for prevention of mother-to-child transmission) [12, 13], over a third of PLHIV have unsuppressed viral load [11], and pre-exposure prophylaxis (PrEP) continues to not be widely available. These all point to the need for using safer conception methods (SCM) such as timed condomless intercourse and manual self-insemination as a compliment to ART. In our prior observational prospective study of PLHIV in Uganda who were trying to conceive, 35% used timed condomless intercourse and just three participants used manual self-insemination, at any time during the 24-month follow-up [14]; however, clients expressed a willingness to use these methods if properly instructed [15]. Our research revealed individual (low SCM awareness; internalized childbearing stigma), partner (HIV non-disclosure; partner willingness to use SCM or attend SCC), and provider (childbearing stigma; lack of SCC knowledge and training) level barriers to SCM use [14, 16].

Our prior research also suggests that HIV-serodiscordant couples who are considering childbearing may decide to delay pregnancy, especially if both members are not certain about wanting a child or if health concerns need to first be resolved [17]. However, studies that have examined fertility desires among PLHIV have often neglected to measure contraception use and intentions. A recent large study found that less than half (43%) of HIV-positive women in Uganda use modern contraceptives; the rate was higher (60%) among those who reported trying to delay or prevent pregnancy [18], but this is still relatively low for achieving their goal of preventing pregnancy.

To help clients and couples achieve optimal reproductive health outcomes, a better understanding is needed with regards to how couples communicate and make decisions about childbearing, including whether or not to have a child as well as the best timing of the pregnancy, and to use either SCM or contraception to achieve what they conclude is their desired pregnancy goal. This knowledge will then better position SCC to help couples progress through these processes that result in decisions and actions towards this goal.

In the context of a larger longitudinal controlled trial of a SCC intervention for Ugandan HIV clients in serodiscordant relationships, we sought to address these important gaps in the literature. Using baseline data, before exposure to any intervention, we explored the following research questions: 1) What differentiates couples who agreed about wanting a child now from couples where at least one member did not want a child now? 2) Among couples who wanted a child now, what differentiates those who had been trying to conceive, from those who had not? 3) Among couples who had been trying to conceive, what differentiates those who report use of SCM from those who report no use of SCM? 4) Among couples who had not been trying to conceive, what differentiates those who report current modern contraception use from those who were not using contraception?

## Methods

### Study setting and design

The study design and protocol, summarized here, has been described in detail in a prior publication [19]. The study took place at six clinics operated by The AIDS Support Organization (TASO). TASO is the oldest and one of the largest indigenous non-governmental organizations in Uganda providing comprehensive HIV care services to HIV infected and affected individuals. Each clinic provides HIV care to 6000–8000 clients, and has a staff of 15–20 medical providers (including three or four family planning nurses). Each clinic was randomly assigned to implement one of two approaches to integrating SCC into family planning services, or usual care (existing family planning services). Based on an ecological adaptation of the Information, Motivation and Behavioral skills (eIMB) model of behavior change [20], we developed a multi-component, structured SCC intervention. The two approaches to implementing the intervention differed on level of intensity of training and supervision of providers.

### Study participants

Clients were eligible if they met the following criteria: 1) in a relationship with an HIV-negative partner (confirmed by HIV test prior to enrollment), 2) of reproductive age (men age 15–60; women age 15–45), 3) considering having a child with their partner, 4) female member of relationship is not currently pregnant (confirmed by a pregnancy test prior to enrollment), and 5) partner is aware of HIV status of client. Recruitment was stratified 50/50 by sex of the client and took place from July 2017 to January 2019. Participants completed assessments at baseline, and months 6 and 12, as well as post-completion of pregnancy (if applicable), but only baseline data were used for this paper’s analysis, as the study is still ongoing. All participants provided written informed consent to participate in the study.

### Measures

The survey included measures of sexual and reproductive health behaviors (use of SCM and contraception), which are described below, and variables that map to our eIMB conceptual framework, as well as other constructs that are potential correlates of the sexual and reproductive health behaviors (see Table 1). The eIMB framework posits that knowledge of why SCM or contraception use is important is essential but often insufficient to change behavior, as motivation and perceived benefit, as well as behavioral skills to engage in the behavior, are key factors. Accordingly, these factors are central to the selection of variables we have examined as correlates of the sexual and reproductive health behaviors we measured. Each measure underwent a translation process in Luganda and Runyakitara (local languages used in the study settings). The survey was interviewer-administered using computer-assisted software (see Supplement materials for copy of survey).

**Table 1:** Measures of eIMB constructs and other potential correlates of sexual and reproductive health behaviors

Construct	Source of measure	Description	Item examples	Response format
**eIMB constructs**				
SCM knowledge	Woldesadik et al. [32]	18-items measure knowledge of availability of SCM in general, specific SCM (e.g., TCI, MSI), and risk reduction strategies not specific to conception (e.g., circumcision, PrEP); number of correct items is summed.	Only having condomless sex during the few days each month when the woman is most fertile helps to limit the risk of HIV transmission to an uninfected partner	True, False, Don’t know
SCM motivation	Gerkovich [33]	2 items measure level of commitment and readiness to use SCM; mean item score is calculated and higher scores reflect greater motivation.	It is important to me that my partner and I use methods that can limit HIV transmission risks during attempts to conceive a child	Strongly agree, agree, disagree, strongly disagree
SCM self-efficacy	Johnson et al. [34]	6 items measure level of confidence to negotiate and utilize SCM; mean item score is calculated and higher scores reflect greater self-efficacy or confidence.	I can follow advice about limiting condomless sex to only 2–3 specific days per month	Strongly agree, agree, disagree, strongly disagree
SCM cultural acceptability	WHO [35]	6 items measure perception of the cultural acceptability of specific SCM. Respondents indicate their level of agreement with statements about the willingness of HIV-affected couples to engage in specific safer conception strategies. Mean item score is calculated and higher scores reflect greater perceived cultural acceptability.	Examples of safer conception strategies enquired about: delaying attempts to conceive until CD4 count is high; use of TCI, MSI, and PrEP	Strongly agree, agree, disagree, strongly disagree
Attitudes towards contraception use		13 items that assess beliefs regarding both positive and negative effects of contraception use. Scoring of negatively worded items is reversed; mean item score is calculated and higher scores reflect more positive attitude towards contraception.	Hormonal contraception can cause permanent sterility in women My religion supports the use of contraception	Strongly agree, agree, disagree, strongly disagree
**Other potential correlates**				
Demographic characteristics	Created in house	Measures of client background characteristics	Age, gender, education level	–
Health management characteristics	Created in house	Information on patient management of HIV disease and immune status that is either self-reported or chart abstracted	Date of HIV diagnosis; most recent CD4 count and HIV viral load; use of HIV antiretroviral therapy	–
Reproductive health history	Created in house	Background on reproductive health history of patient and partner	Number of biological children for patient and partner (including with each other), pregnancy history including miscarriages and abortions	–
**Relationship and partner characteristics**				
Self-agency in decision making within the relationship	Pulerwitz et al. [36]	15-item relationship control measure adapted from Sexual Relationship Power scale to assess self-agency in decision making within the relationship; mean item score is calculated and higher scores reflect greater self-agency.	My partner has more say than I do about important decisions that affect us	Strongly agree, agree, disagree, strongly disagree
Reproductive coercion	Anderson et al. [37]	5-item scale to measure presence of actions from partner to pressure respondent to have a child in the past year; total score is the sum of all items and reflects greater coercion.	Your partner said he/she would leave you if you did not try to have a child	Yes, No
**Provider support for family planning and safer consumption**				
Receipt of family planning counseling	Created in house	A measure to assess whether the client/couple received any consults from their providers in the past 6 months regarding reproductive health decisions and behaviors	Were consults received related to (1) decision to have a child; (2) use of methods to conceive safely; (3) use of contraception to prevent pregnancy	Number of consults received in each category
**Childbearing stigma and pressure**				
Internalized childbearing stigma	Created in house	A single item to measure internalized stigma or shame for wanting to have a child as someone living with HIV	I feel ashamed for wanting a (nother) child	Disagree strongly disagree slightly, neutral, agree slightly, agree strongly
Perceived community stigma towards childbearing	Created in house	3-item measure of perceptions of how family, friends and others in community viewed HIV-affected couples who want to have a child or are pregnant; mean item score is calculated and higher scores reflect greater stigma.	People in the community look down on HIV+ individuals who want to have a child	Disagree strongly disagree slightly, neutral, agree slightly, agree strongly
Provider stigma toward childbearing	Created in house	2-item measure of perceived provider stigma toward childbearing among PLHIV; mean item score is calculated and higher scores reflect greater stigma.	Most HIV providers think that HIV+ clients should not have children	Disagree strongly disagree slightly, neutral, agree slightly, agree strongly
Cultural pressure to have a child		5-item measure of beliefs about how the identify of a man, woman and couple are influenced by whether or not they have children, and perceived expectations from family to have children; mean item score is calculated and higher scores reflect greater perceived cultural pressure.	It is very important that a married couple has children together in order to legitimize the relationship	Strongly disagree, somewhat disagree, somewhat agree, strongly agree
**Psychosocial functioning**				
Internalized HIV stigma	Kalichman et al. [38]	8-item scale measuring stigma about HIV; mean item score is calculated and higher scores reflect greater internalized stigma.	I am ashamed that I am HIV positive	Strongly disagree, disagree, neutral, agree, strongly agree
Depression	Cox et al. [39]	10-item Edinburgh Post-partum Depression Scale to measure depression in past week. The total score is the sum of all items and reflects severity of depressive symptomatology; scores > = 10 reflect possible clinical depression, and scores > 13 reflect likely clinical depression.	In the past 7 days: I have been able to laugh and see the funny side of thing	Response options vary by item but all range from 0 to 3

### Sexual and reproductive health behavior measures

#### Childbearing intentions and recent conception behavior

Participants were asked to indicate whether they want to have a (nother) child, and their perception of their partner’s desire for having a child. Respondents were also asked to indicate (yes/no response) whether they had tried to conceive a child with their partner at any time in the past 6 months.

#### Use of safer conception methods (SCM)

Participants who were trying to conceive were asked whether they used any of these methods to reduce HIV transmission risk to their partner during attempts to conceive in the past 6 months: ***Timed condomless intercourse (TCI)***: “Did you have unprotected or “live” sex only on the two to three specific days each month in which you (your partner) were (was) most fertile?” ***Sperm washing*** (If male respondent): “Did you pay for technology that cleanses your sperm or semen of the HIV virus?” ***Manual self-insemination (MSI)*** (If female respondent): “Did your partner ejaculate into a condom or container and then manually inject the semen into your vagina?” Although not specific to the context of conception, we also asked participants about their partner’s use of pre-exposure prophylaxis (***PrEP)***: “Did your partner take HIV medication every day during the months in which you were trying to conceive?”

To assess correct use of TCI and MSI, respondents were asked in an open-ended format to describe exactly how they carried out the method. The interviewer listened for pre-defined criterion for accurate use (e.g., how the timing of woman’s fertile period was determined; number of days in the fertile period; whether condoms were used each day outside the fertile period; method of collection and insertion of semen, in the case of manual self-insemination). There were six criteria for TCI and eleven for MSI, and the interviewer probed to ascertain whether a specific criterion was met if not spontaneously reported by the respondent. Classification of accurate use required that all criterion be present.

#### Use of contraception

Each respondent was asked if they or their partner currently used contraceptive methods. Male participants were asked to consent to the coordinator calling their female partner at the time of the interview to assess use of female contraceptives (these calls were made in private and responses not shared with the male partner); all but one female partner participated in this assessment. Consistent with Ugandan policy and practice [66], correct use of contraception was defined as using a modern contraceptive (i.e., birth control pills, Depo-Provera (medroxyprogesterone acetate) injection, intrauterine device, implant, sterilization). In addition, we evaluated an alternative definition, that being any use of a modern contraceptive, always using condoms, or sexual abstinence in the past 6 months (as assessed in the measures of sexual behavior described below).

Respondents were first asked to indicate the frequency of sexual intercourse with their partner over the past 6 months; those who reported any intercourse were then asked to report how often condoms were used during intercourse with their partner in the past 6 months. For analysis, a dichotomous variable was created to represent whether or not the participant reported always using condoms. Lastly, we assessed the belief that desire for childbearing impedes consistent condom useby asking participants to respond to the question, “Has the desire for wanting a child contributed to you and your partner not using condoms during intercourse over the past six months?” with a Yes/No response.

### Data analysis

Descriptive statistics were used to describe sample characteristics and the four binary dependent variables being examined: (1) Do both members of the couple want a child now (versus at least one partner not wanting a child right away)? (2) Among those where both partners want a child now, did the couple try to conceive a child in the past 6 months? (3) Among those who were trying to conceive in the past 6 months, was a safer conception method used? And (4) Among those who did not report trying to conceive in the past 6 months, was contraception currently being used? First, bivariate logistic regression models were used to explore associations with all of the potential correlates from our eIMB theoretical model. All variables found to be correlated at *p* < 0.05 with the dependent variable in the bivariate models were included in the multiple logistic regression models to examine the relative contribution of each of these correlates when controlling for the other correlates.

## Results

### Sample description

A sample of 389 PLHIV (195 men, 194 women) who reported considering childbearing with their HIV-negative partner were enrolled in the study. Table 2 lists the sample characteristics with regard to sociodemographics, HIV disease management, reproductive history and relationship with their current partner, psychosocial functioning, utilization of family planning and safer conception-related services, and constructs from our eIMB framework. Mean age was 35.9 years, and two-thirds (66.1%) had only a primary school education or no formal education. Average time since HIV diagnosis was 10.7 years (SD = 8.7 years; range: 1 month to 40 years) and 386 (99.2%) were currently on ART; HIV viral load data was available for 314 (80.7%) participants, of whom 263 (83.8%) had undetectable viral load. Nearly all (*n* = 386; 99.2%) were married to or in a committed relationship with the partner with whom they were considering having a child. Mean duration of the relationship with this partner was 9.9 years (SD = 10.7 years). A large majority (89.7%; *n* = 349) already had biological children, though just half (*n* = 195) had children with their current partner.

**Table 2:** Sample characteristics at baseline

	Mean (SD)/N (%)
**Sociodemographic characteristics**	
Age (years)	35.9 (8.2)
Female gender	194 (49.9%)
Some secondary education	132 (33.9%)
**HIV disease characteristics**	
Time since HIV diagnosis (years)	10.7 (8.7)
CD4 count (cells/mm^3^)	511 (293)
Undetectable HIV viral load (*N* = 315)	264 (83.8%)
Currently on ART	385 (99.0%)
Time on ART (among those on ART, in years)	7.8 (7.1)
**Partner and relationship characteristics**	
Married to partner	326 (83.8%)
Length of relationship (years)	9.9 (10.7)
Currently living with partner	344 (88.4%)
Participant has biological children	349 (89.7%)
Partner has biological children	194 (49.9%)
Has had a child with partner	195 (50.1%)
Self-agency in decision making (PR = 1–4)	3.07 (0.44)
Reproductive coercion (PR = 0–5)	0.13 (0.60)
Discussed childbearing with provider in past 6 months	33 (8.5%)
**Psychosocial functioning**	
Depression (PR = 0–30)	4.21 (4.55)
Possibly clinically depressed	54 (13.9%)
Likely clinically depressed	15 (3.9%)
Internalized HIV stigma (PR = 1–5)	2.19 (0.90)
**eIMB information**	
SCM knowledge (0–18)	11.92 (2.95)
**eIMB motivation**	
SCM motivation	3.73 (0.45)
Positive attitudes towards contraception (PR = 1–4)	2.59 (0.41)
**eIMB behavioral skills**	
SCM self-efficacy	3.47 (0.40)
**eIMB social/ecological**	
Community childbearing stigma (PR = 1–5)	3.27 (1.50)
Provider childbearing stigma (PR = 1–5)	1.92 (0.89)
Internalized childbearing stigma (PR = 1–5)	1.17 (0.60)
SCM cultural acceptability (PR = 1–4)	2.96 (0.59)
**Family planning/SCC services**	
Had any consults with provider about decision to have another child	37 (9.5%)
Had any consults with provider about safer conception methods	22 (5.7%)
Had any consults with provider about contraception	33 (8.5%)

*PR* Potential range of score

### Childbearing intentions and behaviors

Of the 389 PLHIV, 343 (88.2%) said they wanted a child now, 44 (11.3%) said they wanted a child at some point in the future (most within the coming year) and the two remaining respondents said they did not know if they wanted another child. When asked about the desires of their partner, 336 (86.4%) said their partner wanted a child now, 32 (8.2%) said their partner wanted a child at some point in the future, 20 (5.1%) did not know what their partner wanted, and one said their partner did not want another child. When comparing the desires of the participant and their partner, 331 (85.1%) were in agreement in terms of both the desire for and timing of having a child. Most (*n* = 313; 80.5%) of the sample reported that both they and their partner wanted to have a child now. The remaining 76 (19.5%) participants reported that at least one member of the couple did not want to have a child right away.

**Fig. 1:**
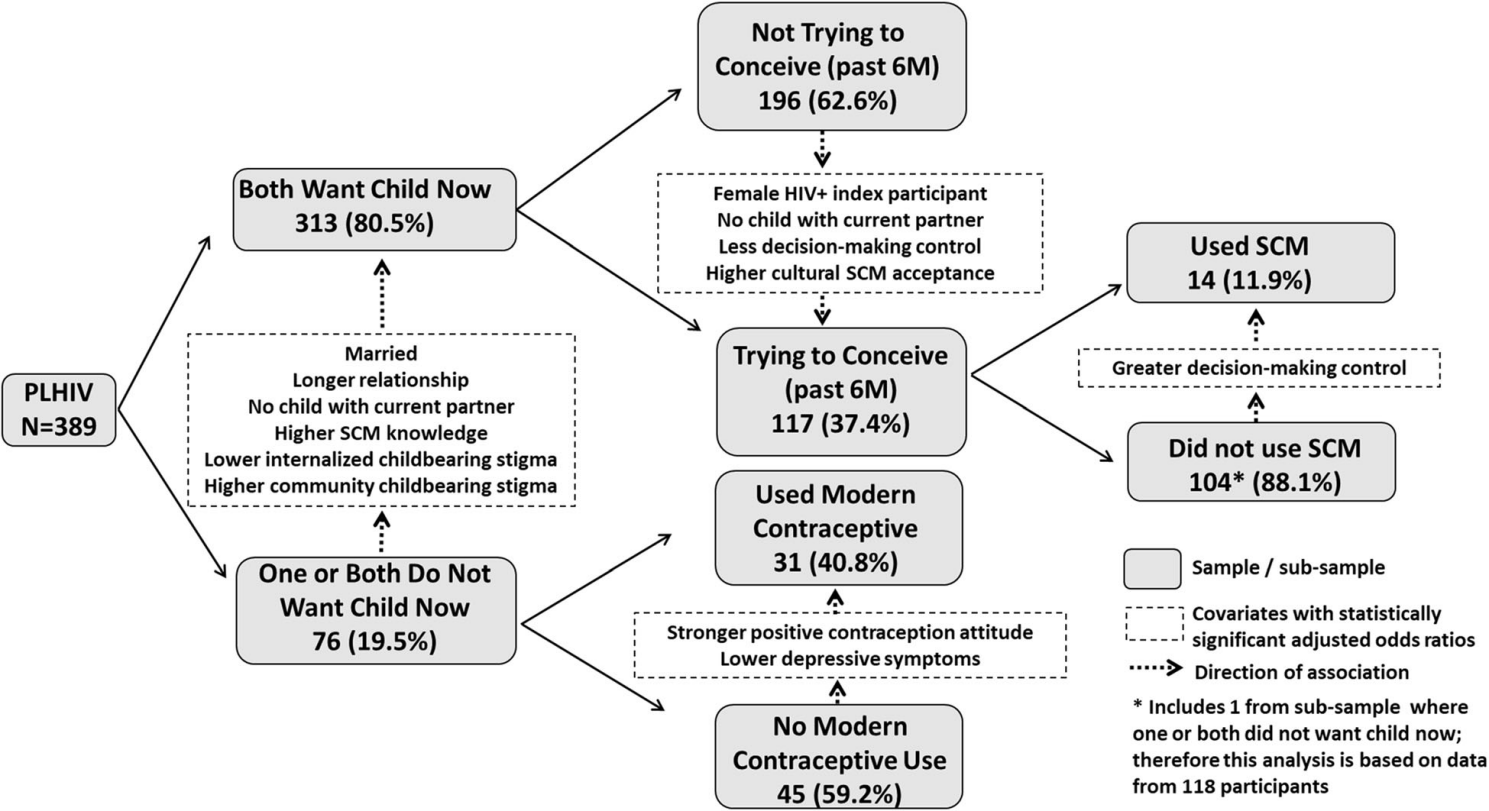
Correlates of key reproductive health intentions and behaviors from multivariate regression analysis

*What differentiates those who report that both they and their partner want a child now, from those who report at least one member of the couple not wanting a child now?* Table 3 lists the bivariate associations with whether or not both members of a couple wanted a child now. In multiple regression analysis, those who reported that both they and their partner wanted a child now were more likely to be married, in a longer relationship, not have a child with their partner, and have greater SCM knowledge and lower internalized childbearing stigma, but higher perceived community stigma of childbearing among PLHIV (see Table 3 and Fig. 1).

**Table 3:** Unadjusted (bivariate) and adjusted (multivariate) logistic regression analysis of correlates of childbearing-related decisions and behaviors

	Full sample (***N*** = 389)		Both members of couple want child now (***N*** = 313)		Tried to conceive in the past 6 months (***N*** = 118)		Did not try to conceive in the past 6 months (***N*** = 268)		
	Both members of couple want child now		Tried to conceive in the past 6 months		Used SCM during attempts to conceive		Current use of modern contraceptives		Use of contraception or behaviors consistent with pregnancy prevention
	UOR (CI)	AOR (CI)^a^	UOR (CI)	AOR (CI)^b^	UOR (CI)	AOR (CI)	UOR (CI)	AOR (CI)	UOR (CI)
**Sociodemographic characteristics**									
Age	1.02 (.99, 1.05)	**–**	0.95*** (.92, .98)	1.03 (.98, 1.07)	0.98 (.91, 1.06)	**–**	0.97 (.94, 1.001)	**–**	1.02 (.99, 1.05)
Female gender	1.13 (.69, 1.87)	***–***	4.16*** (2.54, 6.83)	2.54** (1.28, 5.06)	0.47 (.15, 1.47)	**–**	1.64 (.94, 2.85)	**–**	0.96 (.59, 1.57)
Some secondary education	0.43** (.26, .72)	0.61 (.31, 1.23)	0.66 (.39, 1.10)	**–**	1.76 (.54, 5.73)	**–**	0.94 (.53, 1.66)	**–**	1.12 (.68, 1.85)
**HIV disease characteristics**									
vTime since HIV diagnosis	1.01*** (1.00, 1.01)	1.00 (1.00, 1.01)	1.00 (1.00, 1.00)	**–**	1.00 (1.00, 1.01)	**–**	1.00 (1.00, 1.01)	**–**	1.00 (1.00, 1.00)
Currently on ART	2.07 (.18, 23.09)	**–**	1.00^c^	**–**	0.13 (.01, 2.14)	**–**	1.00^c^	**–**	1.00^c^
CD4 count^d^	1.00 (1.00, 1.00)	**–**	1.002** (1.001,1.003)	**−**^h^	1.00 (1.00, 1.00)		1.002* (1.000, 1.003)	**−**^**f**^	1.00 (1.00, 1.00)
Undetectable HIV viral load^e^	***3.52 (1.81, 6.83)***	**−**^g^	1.08 (.50, 2.36)		0.56 (.10, 2.98)		1.04 (.47, 2.29)		1.57 (.79, 3.12)
Time on ART	1.01*** (1.01, 1.01)	**–**	1.00 (.99, 1.00)	**–**	1.00 (.99, 1.01)	**–**	1.00 (1.00, 1.00)	**–**	1.00 (1.00, 1.00)
**Partner and relationship characteristics**									
Married to partner	3.55*** (1.97, 6.38)	4.96*** (1.75, 14.04)	1.35 (.65, 2.78)	**–**	1.00^c^	**–**	0.63 (.33, 1.22)	**–**	1.02 (.55, 1.88)
Length of relationship	1.06** (1.02, 1.09)	1.08* (1.02, 1.15)	0.94*** (.91, .96)	0.99 (.96, 1.03)	1.03 (.97, 1.08)	**–**	0.96* (.93, .99)	0.96* (.93, .99)	1.00 (.98, 1.02)
Currently living with partner	2.93** (1.51, 5.70)	1.02 (.36, 2.93)	1.30 (.57, 2.97)	**–**	1.00^c^	**–**	1.05 (.47, 2.35)	**–**	0.99 (.49, 2.00)
Participant has biological children	0.31 (.09, 1.02)	–	0.33*** (.16, .68)	0.46 (.19, 1.11)	0.52 (.15, 1.86)	**–**	2.54 (.56, 11.45)	**–**	4.00* (1.26, 12.74)
Partner has biological children	1.06 (.64, 1.75)	**–**	2.77*** (1.72, 4.46)	1.61 (.90, 2.89)	0.65 (.21, 2.01)	**–**	1.45 (.83, 2.51)	**–**	1.14 (.70, 1.84)
Has had a child with partner	0.33*** (.19, .58)	0.09*** (.04, .21)	0.19*** (.11, .31)	0.33** (.16, .67)	0.96 (.25, 3.73)	**–**	1.38 (.77, 2.46)	**–**	1.49 (.91, 2.46)
Self-agency in decision making	2.02* (1.13, 3.64)	1.58 (.74, 3.38)	0.19*** (.10, .35)	0.39* (.19, .81)	6.34*** (1.64, 24.50)	5.80* (1.27, 26.50)	0.37** (.18, .73)	0.34** (.16, .74)	0.69 (.38, 1.24)
Reproductive coercion	0.95 (.63, 1.41)	**–**	1.32 (.87, 1.99)	**–**	1.00^c^	**–**	1.41 (.91, 2.19)	**–**	1.31 (.79, 2.16)
Discussed childbearing with provider	1.10 (.44, 2.77)	**–**	2.07 (.92, 4.65)	**–**	5.86*** (1.62, 21.29)	5.29 (.95, 29.42)	0.65 (.18, 2.35)	**–**	1.85 (.62, 5.47)
**Psychosocial functioning**									
Depression	0.97 (.92, 1.02)	**–**	1.11*** (1.05, 1.17)	1.04 (.97, 1.11)	1.02 (.91, 1.15)	**–**	0.95 (.88, 1.01)	***–***	0.98 (.93, 1.03)
Possibly clinically depressed	0.73 (.37, 1.44)	**–**	2.15* (1.11, 4.16)	**–**	1.91 (.54, 6.78)	**–**	0.27* (.08, .91)	0.20* (.06, .72)	0.79 (.38, 1.65)
Likely clinically depressed	0.35 (.12, 1.00)	–	3.46 (.85, 14.11)	**–**	1.00^c^	**–**	1.00^c^	**–**	0.39 (.10, 1.61)
Internalized HIV stigma	0.57*** (.43, .75)	0.83 (.54, 1.29)	1.21 (.94, 1.56)	**–**	1.04 (.56, 1.90)	**–**	1.13 (.83, 1.53)	**–**	0.98 (.75, 1.28)
**eIMB information**									
SCM knowledge	1.21*** (1.11, 1.32)	1.18* (1.04, 1.33)	0.91* (.84, .99)	0.96 (.87, 1.06)	1.03 (.84, 1.27)	**–**	1.04 (.95, 1.15)	–	1.01 (.93, 1.09)
**eIMB motivation**									
SCM motivation	**–**	**–**	**–**	**–**	2.62 (.49, 14.19)	**–**	**–**	**–**	**–**
Positive attitudes towards contraception	1.20 (.65, 2.22)	**–**	0.53* (.31, .92)	0.63 (.32, 1.25)	–	**–**	2.13* (1.09, 4.19)	3.17** (1.47, 6.83)	1.08 (.61, 1.91)
**eIMB behavioral skills**									
SCM self-efficacy	–	**–**	**–**	**–**	7.42* (1.30, 42.24)	5.16 (.91, 29.27)	**–**	**–**	**–**
**eIMB social/ecological**									
Community childbearing stigma	1.37*** (1.15, 1.63)	1.40* (1.05, 1.76)	0.98 (.84, 1.13)	**–**	0.72 (.49, 1.06)	**–**	0.96 (.80, 1.16)	**–**	0.97 (.83, 1.14)
Provider childbearing stigma	0.96 (.72, 1.28)	**–**	1.18 (.92, 1.52)	**–**	1.30 (.70, 2.42)	**–**	0.76 (.54, 1.07)	**–**	0.96 (.73, 1.26)
Internalized childbearing stigma	0.61** (.43, .87)	0.44** (.28, .71)	0.72 (.42, 1.22)	**–**	1.00^c^	**–**	0.93 (.61, 1.41)	**–**	1.41 (.93, 2.12)
SCM cultural acceptability	0.51** (.32, .80)	0.65 (.34, 1.25)	1.74** (1.17, 2.59)	1.92* (1.19, 3.08)	1.72 (.60, 4.93)	**–**	1.29 (.81, 2.06)	**–**	1.41 (.93, 2.13)
**FP/SCC services**									
Had any consults with provider about decision to have another child	1.04 (.44, 2.48)	**–**	1.90 (.88, 4.10)	**–**	***–***	**–**	0.70 (.23, 2.18)	**–**	2.59 (.92, 7.35)
Had any consults with provider about safer conception methods	1.57 (.45, 5.46)	**–**	1.93 (.76, 4.90)	**–**	6.53*** (1.58, 27.10)	1.05 (.23, 4.85)	**–**	**–**	–
Had any consults with provider about contraception	1.10 (.44, 2.77)	**–**	1.05 (.46, 2.39)	**–**	***–***	**–**	0.28 (.06, 1.25)	**–**	1.69 (.66, 4.32)

*** *p* < .001 ** *p* < 0.01, * *p* < 0.05

^a^This multiple regression model did not include time on ART because it was highly correlated (*r* = .84) with time since HIV diagnosis

^b^This multiple regression model included depressive symptomatology, but not the likely presence of a depressive disorder due to the lack of independence between these two variables

^c^A standard error and confidence interval could not be computed because one or both groups had all participants with the same response value

^d^CD4 count data were available for 252 participants at baseline

^e^HIV viral load data were available for 315 participants at study baseline

^f^CD4 count was not included in this reported multiple regression model because only 167 of 268 participants had CD4 data

^g^Viral load was not included in the multiple regression model presented in this table because of the high number of cases with missing data; when viral load was added to the multiple regression model, it was not significantly correlated with the dependent variable when adjusting for the other variables that were significant bivariate correlates

^h^CD4 count was not included in the multiple regression model presented in this table because of the high number of cases with missing data; when CD4 count was added to the multiple regression model, it remained significantly correlated with the dependent variable when adjusting for the other variables that were significant bivariate correlates

*Among those who reported that both they and their partner wanted a child now, what differentiates those who had been trying to conceive, from those who had not?* Among the 313 who reported that both they and their partner wanted a child now, 117 (37.4%) reported that they and their partner had been trying to conceive in the prior 6 months, while the others had not been trying to conceive. Table 3 lists the bivariate associations with recent attempts to conceive. In multiple regression analysis, those who had been trying to conceive were more likely to be female, not have had a child with their current partner, report less decision-making control within the relationship, and greater perceived cultural acceptability of SCM (see Table 3 and Fig. 1).

*Among those trying to conceive, what differentiates those who report use of SCM from those who report no use of SCM?* Of the 118 who had tried to conceive in the past 6 months (the 117 described above, plus one additional participant who reported that they did not want a child now but their partner did), only 14 (11.9%) reported using a SCM, with nearly all (*n* = 13) using timed condomless intercourse and just one using manual self-insemination. Follow-up questions asking participants to describe the exact steps they used to implement the method revealed that none were using the methods correctly. None reported that their partner used PrEP during the time they were trying to conceive. Other strategies used to reduce transmission risk and/or ensure that female partners were ready for pregnancy included delaying attempts to conceive until a higher CD4 count (*n* = 22) or undetectable viral load (*n* = 31) was achieved, starting ART earlier than they would have otherwise (*n* = 15), seeking testing and treatment (if needed) for sexually transmitted infections (*n* = 20), and male partner circumcision (*n* = 52). The vast majority (93.2%; *n* = 110) reported never (*n* = 84) or sometimes (*n* = 26) using condoms during sexual intercourse with their partner in the past 6 months, and 90 (76.3%) said that the desire to have a child contributed to their lack of condom use.

Table 3 lists the bivariate correlates of SCM use in the 6 months prior to baseline among those who reported trying to conceive. It is particularly noteworthy that rates of full viral suppression (i.e., undetectable HIV viral load) did not vary significantly between those who had used SCM (81.8%) and those who did not use SCM (89.0%; FET = .61). In the multiple regression analysis, the only independent correlate of SCM use was self-agency in decision making within the relationship, as those who used SCM had greater control in decision making, compared to those who did not use SCM (see Table 3 and Fig. 1).

*Among those who were not trying to conceive, what differentiates those using modern contraception from those who were not using contraception?* Of the 268 participants who reported not trying to conceive with their partner in the past 6 months, just one in four (*n* = 69; 25.7%) reported that the female partner was using a modern contraceptive: 31 used Depo-Provera injections, 25 had an implant, three used birth control pills and 10 had an intrauterine device. Table 3 lists the bivariate associations with use of modern contraceptives in this subgroup. In the multiple regression models, those using modern contraceptives were more likely to have been in a relationship with their partner for a shorter time period, have less control over decision making within the relationship, stronger positive attitudes towards contraception and less likely to have depression (see Table 3 and Fig. 1).

All but two of these 268 participants provided data on sexual intercourse with their partner over the past 6 months; 260 reported having sexual intercourse with their partner, while six reported no intercourse with their partner (one of whom also reported use of a modern contraceptive). Of the 260 who had any intercourse, 67.3% reported never (*n* = 109) or sometimes (*n* = 66) using condoms with their partner during this period, while 85 reported always using condoms (including 11 who were using modern contraceptives). Just over half (*n* = 148; 55.2% of the subgroup of 268) reported either using a modern contraceptive, always using condoms, or sexual abstinence in the 6 months prior to baseline. Participants who already had a biological child of their own were more than four times more likely to report a behavior consistent with trying to avoid pregnancy, compared to those who were not using modern contraceptives, consistent condom use or abstinence (see Table 3 and Fig. 1); there were no other significant bivariate correlates.

## Discussion

In this sample of HIV clients who reported considering having a child at enrollment, the vast majority reported that both they and their partner wanted to have a child right away. Nevertheless, less than a third of these clients had recently been trying to conceive. Among those who had been trying to conceive, just 1 in 10 were using SCM, with timed condomless intercourse being almost the sole method used. Conversely, among those who reported not trying to conceive, only one quarter reported current use of modern contraceptives, while just over half reported using some type of method (contraceptives, consistent condom use, abstinence) to prevent pregnancy. Relationship characteristics and perceived community attitudes towards pregnancy among PLHIV were among the independent correlates of intentions and behaviors aimed at conception, while self-agency in decision making was an independent correlate of use of both SCM and contraception. Individual-level constructs related to the ecological adaptation of the Information Motivation and Behavioral skills model of health behavior were not prominent as correlates of these reproductive decisions and behaviors, particularly when controlling for other variables associated with the behavior.

Most but not all reported agreement between themselves and their partner on the desire for and timing of pregnancy. Participants who were married to their partner, been together with their partner longer, and had no prior children with their partner, were more likely to report that both they and their partner wanted a child now, suggesting that those in stable relationships are more likely to be motivated to want a child sooner rather than later. This appears to be especially true when they have had no prior children together, which culturally is considered to be important for legitimizing and securing a relationship in Uganda [20–22].

Participants who reported that both they and their partner wanted a child now also had greater SCM knowledge, and less internalized stigma about childbearing, despite perceiving greater stigma from the community regarding childbearing among PLHIV. These findings suggest that having more information about how to have a safe, healthy pregnancy and not feeling shame or stigma about wanting a child as someone living with HIV, enables a couple to feel empowered to want to have a child, even in the face of perceived stigma in the community. Furthermore, having greater SCM knowledge may serve as a buffer against the effects of external stigma, as such couples may understand that they are able to prevent transmission of HIV to the child and partner, as this is what likely drives community concerns about pregnancy in this population. The greater perceived stigma in the community may also be a biproduct of lower internalized stigma and shame which may lead one to talk more about wanting and trying to have a child, which may increase exposure to stigmatizing views from others. Couples living with HIV, perhaps especially those in serodiscordant relationships who are yet to have children together, are likely weighing the opposing pressures of needing to have children to legitimize their marriage (particularly if they have not disclosed their HIV status to family and friends), and wanting to avoid stigma and shame from others about having a child and risking transmission of HIV to both the child and uninfected partner [23]. Which of these pressures is most influential will vary across couples, and may have played a role in our observation that a minority of couples who wanted to have a child were actually trying to conceive.

Although the vast majority said both they and their partner wanted a child now, just under a third of such respondents said they had been trying to conceive in the months prior to enrollment. Trying to conceive was associated with the index participant (and HIV-positive member of the couple) being female, and having less agency in decision making within the relationship. In Uganda, cultural norms dictate that women have less control over decisions [20], and this may extend to decisions and behaviors related to sex and childbearing [24], so women are more vulnerable to being pressured by their partners to try to conceive [20]. The fact that all the women (whether they were an index participant or HIV-negative partner) in this part of the analysis were indicated as having a desire to have a child now, would seem to negate the potential for the woman to be pressured by the man to seek childbearing, but it is possible that some of these women felt pressured either by their partner or others (e.g., family, community) to express a desire to have a child now [2, 8, 25]. Not having prior children with one’s current partner was another independent correlate of trying to conceive; as stated above, this is a clear motivator for not only wanting a child now, but also actively trying to conceive. Trying to conceive was also associated with greater perceived cultural acceptability of SCM in the community, which suggests that perceived support or acceptance for strategies to ensure a healthy, safe pregnancy among PLHIV may help a couple to decide to move forward with trying to conceive.

Among those trying to conceive, just 1 in 10 were using SCM, with timed condomless intercourse being the method used most. Important to note is that not a single participant reported implementing these methods accurately. Knowing how to determine the timing of the 3-day fertile period for the woman seemed to be the most challenging aspect for implementing timed condomless intercourse and manual self-insemination. These findings are likely in part a reflection of the general low knowledge of SCM and how to properly implement the methods, as also found in our prior research [15–17]. Use of SCM was associated with having more self-agency or control in decision making within the relationship, which suggests that SCM are more likely to be used if the members of the couple feel they have a say in their childbearing decision process. Safer conception counseling (SCC) helps to promote autonomy and self-agency in the context of childbearing decisions by facilitating an informed decision making process in which the couple receives information and instruction about what contributes to a healthy pregnancy, and increased access to SCM by informing couples about timed condomless intercourse and manual self-insemination, as well as support to implement these methods [26]. Our prior qualitative research suggests that these couples are willing to use these methods with proper instruction [15, 17], further highlighting the need for SCC to ensure uptake and effective use of SCM.

Among those who reported not trying to conceive in the months leading up to baseline, just one in four reported using modern contraceptives at the time of the baseline interview. This is much lower than the 68% found in a recent large study of HIV-positive women in Uganda who were seeking to prevent pregnancy [18]. While the respondent and their partner may not have yet been in a position to agree on actively pursuing pregnancy prior to baseline, some level of desire to have a child was present within the relationship (given our enrollment criteria). This desire may serve as a strong barrier to contraception use, particularly in the larger context of cultural pressure to have children. Being with one’s partner for a shorter period of time was associated with using modern contraception, while those who already had biological children were much more likely to be using some method (contraception, condoms, abstinence) to prevent pregnancy, further highlighting how one’s relationship context can influence reproductive behaviors.

Use of modern contraceptives was also associated with lower depression and greater positive attitudes towards contraception [27]. Better mental health and psychological well-being is conducive to making lucid, coherent decisions and better health behavior, as well as motivation to engage in behavior that is consistent with one’s goals [28], including contraception to prevent pregnancy [27]. Unfortunately, mental health problems, including depression, largely go undetected and untreated in low resource settings, including Uganda [29]. Greater priority on depression screening and treatment is needed in all health settings, but especially in HIV primary care and family planning for the population targeted in this study. These findings also suggest the importance of having positive attitudes and beliefs regarding modern contraceptives, which is likely related to greater knowledge and understanding about how modern contraceptives work and the importance of receiving quality instruction and support from family planning providers. Misconceptions and myths about modern contraceptives are not uncommon in Uganda and the larger region of sub-Saharan Africa [30, 31], as well as other barriers such as stock outs, and health centers often not having all contraceptive types (e.g., intrauterine device, long acting reversible contraceptives). These are all factors that must be addressed to increase uptake and proper use of modern contraceptives.

While a minority of those not trying to conceive right away were using modern contraceptives, just over half (55%) were either using modern contraceptives or consistent condom use (or abstinence). However, given the risk of HIV transmission to the uninfected partner, it is noteworthy that roughly two-thirds of these participants reported inconsistent condom use with their partner, including many who never use condoms. Most of the sample had undetectable viral loads at their last viral load testing, which provides protection against transmission risks, but some level of risk for transmission remains given the uncertain presence of spikes in viremia in between the annual viral load tests that are done as part of usual care. Inconsistent condom use was present, despite the intention to prevent pregnancy, although a desire for children now or in the future was present for most participants or their partners, and this was likely a contributing factor to lack of condom use.

Limitations to this study include the absence of direct data collected from the partner of the index participant, except for female partners of male participants who were briefly interviewed to collect information regarding contraception use. Our data largely relies on the perceptions of the index participant with regards to the attitudes and behaviors of the partner. Other limitations include the reliance on self-reported data, including use of SCM and contraceptives, the cross-sectional nature of the data, and the sample being comprised solely of PLHIV who are receiving HIV care. Clinic data related to prescription of contraceptives would strengthen our data regarding contraception use. The cross-sectional nature of the data enables us to examine associations but not causality or even temporal inferences, the latter of which may be possible with the longitudinal data to be collected in this study. With the sample being limited to those in care, our findings may not reflect PLHIV who are not in HIV care and perhaps less likely to be familiar with safer conception and contraception methods and how to use them.

## Conclusion

Our findings offer suggestions for how health services can better support the desired pregnancy goals of clients and couples affected by HIV. First, providers need to understand what clients and their partners have discussed and how they have made their current childbearing decisions, including consideration of past attempts to conceive. Providers need to recognize the influence of partners and the relationship on these decisions, as well as the impact of perceived community attitudes towards PLHIV having children. They also need to take into account the sex of the HIV-positive partner and self-agency in relationship decision making as potential drivers of childbearing decisions and behaviors. SCC is a process through which counselors can facilitate couple communication and decision making regarding the best timing of the pregnancy, as well as use of safer conception or contraceptive methods to achieve their desired reproductive outcome. SCC is an opportunity to empower couples to make informed decisions and take appropriate actions to achieve their desired goals, and such counseling can promote both members of the couple having a say in their childbearing decisions, provide information that can address negative attitudes towards contraceptives, and facilitate access to services that can address mental health needs. Finally, given the influence of perceived stigma in the community towards childbearing among PLHIV, it is important to educate communities on the reproductive rights and choices available to HIV affected couples and the clinic support services that can facilitate safer conception to minimize the risk of HIV transmission to either the child or the uninfected partner.

## Supplementary Information

**Additional file 1.**

## Data Availability

An anonymous dataset is available to researchers upon request to the corresponding author and review by the study team.

## References

[1] Kanyangarara M, Sakyi K, Laar A (2019). Availability of integrated family planning services in HIV care and support sites in sub–Saharan Africa: a secondary analysis of national health facility surveys. Reprod Health.

[2] Beyeza–Kashesya J (2010). My partner wants a child: a cross–sectional study of the determinants of the desire for children among mutually disclosed sero–discordant couples receiving care in Uganda. BMC Public Health.

[3] Beyeza–Kashesya J (2011). To use or not to use a condom: a prospective cohort study comparing contraceptive practices among HIV–infected and HIV–negative youth in Uganda. BMC Infect Dis.

[4] Heys J (2009). Fertility desires and infection with the HIV: results from a survey in rural Uganda. AIDS.

[5] Kakaire O, Osinde MO, Kaye DK (2010). Factors that predict fertility desires for people living with HIV infection at a support and treatment Centre in Kabale, *Uganda*. Reprod Health.

[6] Wagner G (2012). Factors associated with intention to conceive and its communication to providers among HIV clients in Uganda. Matern Child Health J.

[7] Wanyenze RK (2011). Uptake of family planning methods and unplanned pregnancies among HIV–infected individuals: a cross–sectional survey among clients at HIV clinics in Uganda. J Int AIDS Soc.

[8] Beyeza–Kashesya J (2009). The dilemma of safe sex and having children: challenges facing HIV sero–discordant couples in Uganda. Afr Health Sci.

[9] Wabwire–Mangen F (2009). Uganda HIV prevention response and modes of transmission analysis.

[10] Cohen MS (2011). Prevention of HIV–1 infection with early antiretroviral therapy. N Engl J Med.

[11] UNAIDS (2020). Uganda: overview.

[12] Mills EJ (2006). Adherence to HAART: a systematic review of developed and developing nation patient–reported barriers and facilitators. PLoS Med.

[13] UNAIDS (2013). HIV and AIDS Uganda country Progress report.

[14] Wagner GJ (2015). Correlates of use of timed unprotected intercourse to reduce horizontal transmission among Ugandan HIV clients with fertility intentions. AIDS Behav.

[15] Goggin K (2014). “Our hands are tied up”: current state of safer conception services suggests the need for an integrated care model. Health Care Women Int.

[16] Wagner GJ (2017). Prevalence and correlates of use of safer conception methods in a prospective cohort of Ugandan HIV–affected couples with fertility intentions. AIDS Behav.

[17] Mindry D (2017). Safer conception for couples affected by HIV: structural and cultural considerations in the delivery of safer conception Care in Uganda. AIDS Behav.

[18] Wanyeze R (2017). Family planning and sexual and reproductive health survey among HIV infected individuals in HIV Care in Uganda.

[19] Goggin K (2018). Study protocol of “our choice”: a randomized controlled trial of the integration of safer conception counseling to transform HIV family planning services in Uganda. Implement Sci.

[20] Agol D (2014). Marriage, intimacy and risk of HIV infection in south West Uganda. Afr J Reprod Health.

[21] Kabagenyi A (2016). Socio–cultural inhibitors to use of modern contraceptive techniques in rural Uganda: a qualitative study. Pan Afr Med J.

[22] Ngure K (2013). My intention was a child but I was very afraid: fertility intentions and HIV risk perceptions among HIV–serodiscordant couples experiencing pregnancy in Kenya. AIDS Care.

[23] Kisakye P, Akena WO, Kaye DK (2010). Pregnancy decisions among HIV–positive pregnant women in Mulago hospital, Uganda. Cult Health Sex.

[24] Derose L, Ezeh A (2010). Decision–making patterns and contraceptive use: evidence from Uganda. Popul Res Policy Rev.

[25] Airhihenbuwa CO, Webster JD (2004). Culture and African contexts of HIV/AIDS prevention, care and support. SAHARA J.

[26] Mindry D (2018). Benefits and challenges of safer–conception counseling for HIV Serodiscordant couples in Uganda. Int Perspect Sex Reprod Health.

[27] Lasater ME (2017). Addressing the unmet need for maternal mental health services in low– and middle–income countries: integrating mental health into maternal health care. J Midwifery Womens Health.

[28] Parletta N, Aljeesh Y, Baune BT (2016). Health behaviors, knowledge, life satisfaction, and wellbeing in people with mental illness across four countries and comparisons with normative sample. Front Psychiatry.

[29] Collins PY (2006). What is the relevance of mental health to HIV/AIDS care and treatment programs in developing countries? A systematic review. AIDS.

[30] Gueye A (2015). Belief in family planning myths at the individual and community levels and modern contraceptive use in urban Africa. Int Perspect Sex Reprod Health.

[31] Thummalachetty N (2017). Contraceptive knowledge, perceptions, and concerns among men in Uganda. BMC Public Health.

[32] Woldetsadik MA, et al. Safer Conception Methods and Counseling: Psychometric Evaluation of New Measures of Attitudes and Beliefs Among HIV Clients and Providers. AIDS Behav, 2016;20(6):1370–81. 10.1007/s10461-015-1199-3 PMC5537001 26487299

[33] Gerkovich M, et al. Gerkovich M, Williams K, Catley D, Goggin K, in IAPAC. Miami; 2008.

[34] Johnson M, et al. The Role of Self–Efficacy in HIV Treatment Adherence: Validation of the HIV Treatment Adherence Self–Efficacy Scale (HIVASES). J Behav Med, 2007. 10.1007/s10865-007-9118-3 PMC2423379 17588200

[35] World Health Organization. User Preferences for Contraceptive Methods in India, Korea, the Philippines and Turkey. Stud Fam Plan. 1980;11(9/10):268–73. 6213071

[36] Pulerwitz J, Gortmaker S, DeJong W. Measuring Relationship Power in HIV/STD Research. Sex Roles. 2000;42(7/8).

[37] Anderson J, Grace K, Miller E. Reproductive coercion among women living with HIV: an unexplored risk factor for negative sexual and mental health outcomes.. AIDS. 2017;31. 10.1097/QAD.0000000000001620 PMC5633520 28832408

[38] Kalichman S, et al. Development of a brief scale to measure AIDS–related stigma in South Africa. .AIDS Behav, 2005;9(2):135–43. 10.1007/s10461-005-3895-x 15933833

[39] Cox J, Holden J, Sagovsky R, Detection of postnatal depression. Development of the 10–item Edinburgh Postnatal Depression Scale. Br J Psychiatry. 1987;150:782–6. 10.1192/bjp.150.6.782 3651732

