# Supplementary material for: Reproductive intentions and corresponding use of safer conception methods and contraception among Ugandan HIV clients in serodiscordant relationships
Source: BMC Public Health. 2021 Jan 19;21:156. doi: 10.1186/s12889-021-10163-7 (PMC7814634; doi:10.1186/s12889-021-10163-7)
Supplement: Supplementary file 1 — Additional file 1. [file 12889_2021_10163_MOESM1_ESM.rtf]

cBR Survey (English)Current date and time is recorded [TODAY] at [STTIME].   If this is not correct exit and correct device time.Preliminary InformationP1.	Participant ID:	__ __ __ __P2.	Site ID:  (Choose one)	1	Jinja	2	Mbale	3	Entebbe	4	Masaka	5	Mbarara	6	RunkungiriP3.	ASSESSMENT:  (Choose one)	1	Baseline	2	Month 6	3	Month 12	4	Post-PregnancyP4.	Interviewer Initials:	__ __P5.	Participant Initials:	__ __P6.	Gender of participant:	1	Male	2	FemaleP7.	Is female partner available to respond to contraception questions:  (Choose one)	0	No	1	Yes	2	Not applicable, as respondent is female	3	Not applicable, as male respondent is now single	8	Refuse to AnswerThank you for agreeing to participate in this study. We truly value your time and contribution to this study and its effort to improve the reproductive needs of clients living with HIV. This survey will take around 60 minutes to complete. All information you provide will be kept confidential and not shared with anyone else.  Please let me know if you have a question at any point throughout the survey.If P3 is not equal to 1, then skip to B3.Demographics (Baseline only)I will begin by asking you a few background questions.A1.	What is your age?	__ __A2.	What is the highest level of school you attended?  (Choose one)	0	No formal schooling	1	Some primary	2	Completed primary	3	Some secondary	4	Completed secondary	5	Any university	6	Other post-secondary tertiary	8	Refuse to AnswerA3.	What is your religion?  (Choose one)	1	Catholic	2	Protestant	3	Muslim	4	Pentecostal	5	Seventh Day Adventist	6	Other	8	Refuse to AnswerA4.	What is your primary occupation?  (Choose one)	1	Peasant farmer	2	Salaried	3	Business/sell things	4	Casual worker	5	Other	8	Refuse to AnswerA5.	How much time does it take to travel round trip from your home to this clinic?	__ __ __	minutes	998	Refuse to AnswerA6.	What does it cost for the round trip to the clinic and back?	__ __ __ __ __ __ __	Ush	9999998	Refuse to AnswerA7.	Do you sometimes worry that you won't have the money to get to the clinic for a scheduled visit?	1	Yes	0	No	8	Refuse to AnswerHIV disease and medical health characteristicsThe questions below ask about your HIV disease and HIV care.B1.	How long ago did you learn that you were HIV+?	__ __ __	YEARS	__ __ __	MONTHS	998	Refuse to Answer (Months)If B1Y is greater than A1 then Number of years cannot be greater than age (&[AGE]). and skip to B1.B2.	How long ago did you start receiving HIV care, either at this clinic or somewhere else?	__ __ __	YEARS	__ __ __	MONTHS	998	Refuse to Answer (Months)If B2 is greater than B1 then Time since start of care cannot be greater than time since learning you were HIV+ (&[B1] ago). and skip to B2.B3.	Are you currently taking antiretroviral drugs (ARVs) to manage your HIV?	1	Yes	0	No	8	Refuse to AnswerIf B3 is equal to 0 or B3 is equal to "Refuse to Answer", then skip to B5a.B4a.	How long ago did you first start taking ARVs to manage your HIV?	__ __ __	YEARS	__ __ __	MONTHS	998	Refuse to Answer (Months)If B4a is greater than B2 and B2 is not equal to "skipped" then Time since start of taking ARVs to manage your HIV cannot be greater than time since start of care (&[B2] ago). and skip to B4a.We'd like to know about how you have been managing your HIV medications over the last several days. Don't worry about telling us that you don't always take all of your medication. Most people are not able to take every pill as directed. It is important for us to understand the difficulties that people may be having with taking their HIV medication.B4b.	How many ARV doses are you supposed to take each day?	__	doses	8	Refuse to AnswerB4c.	How many ARV doses did you miss in the LAST 7 DAYS?	__ __	doses	98	Refuse to AnswerB5a.	In the past six months, how many scheduled appointments did you have with your HIV provider as part of your regular HIV care?	__ __	98	Refuse to AnswerB5b.	How many of these appointments did you attend?	__ __	98	Refuse to AnswerIf B5b is greater than B5a then Number of appointments attended cannnot be greater than number of appointments scheduled (&[B5A]). and skip to B5b.If P3 is equal to 1, then skip to instruction before C1.B6.	Are you still in a relationship with the partner that you were with at our last study interview?  (Choose one)	0	No, and I am not in a relationship with anyone now	1	No, but I am in a relationship with someone else	2	Yes, I am still in a relationship with that person	8	Refuse to AnswerIf B6 is equal to 2 or B6 is equal to 8, then skip to C4.B6_1.	Was the ending of the relationship influenced by the two of you disagreeing about trying to have a child?  (Choose one)	0	No, this was not a factor in the break-up	1	Yes it was one factor in the break-up, but there were other reasons as well	2	Yes it was the main reason for the break-up	5	I don't know whether this was a reason for the break-up	8	Refuse to AnswerB6_2.	Was the ending of the relationship influenced by you and your partner having a hard time conceiving?  (Choose one)	0	No, this was not a factor in the break-up	1	Yes it was one factor in the break-up, but there were other reasons as well	2	Yes it was the main reason for the break-up	5	I don't know whether this was a reason for the break-up	8	Refuse to AnswerIf B6_1 is not equal to 1 and B6_1 is not equal to 2 and B6_2 is not equal to 1 and B6_2 is not equal to 2, then skip to B6a.B6_3.	Can you tell me a bit about this? When did you start to consider ending the relationship?  Did the status of your relationship change once you got in the study or met with the providers to learn about safer conception.	__ __ __ __ __ __ __ __ __ __ __ __ __ __ __ __ __ __ __ __ __ __ __ __ __ __ __ __ __ __ __ __ __ __ __ __ __ __ __ __ __ __ __ __ __ __ __ __ __ __ __ __ __ __ __ __ __ __ __ __ __ __ __ __ __ __ __ __ __ __ __ __ __ __ __ __ __ __ __ __ __ __ __ __ __ __ __ __ __ __ __ __ __ __ __ __ __ __ __ __ __ __ __ __ __ __ __ __ __ __ __ __ __ __ __ __ __ __ __ __ __ __ __ __ __ __ __ __ __ __ __ __ __ __ __ __ __ __ __ __ __ __ __ __ __ __ __ __ __ __ __ __ __ __ __ __ __ __ __ __ __ __ __ __ __ __ __ __ __ __ __ __ __ __ __ __ __ __ __ __ __ __ __ __ __ __ __ __ __ __ __ __ __ __ __ __ __ __ __ __B6a.	Since the last study interview, have you had a pregnancy with the partner you had in the last interview?	1	Yes	0	No	8	Refuse to AnswerIf B6 is equal to 0 and B6a is equal to 0 then skip to instruction before C1If not, then skip to instruction before C1.For the remaining questions that pertain to your partner, consider the partner you had when you started the study.If B6 is equal to 0 and B6a is equal to 0, then skip to C7a.If (B6 is equal to 0 or B6 is equal to 1) and B6a is equal to 1 then skip to instruction before C1If not, then skip to instruction before C1.For the remaining questions that pertain to your partner, consider the partner you had when you started the study, NOT your current partner.If (B6 is equal to 0 or B6 is equal to 1) and B6a is equal to 1, then skip to C7a.Partner/relationship characteristicsIf P3 is equal to 1 then skip to instruction before C1If not, then skip to instruction before C1.The next few questions are related to your relationship with your partner with whom you are considering having a child. If you have multiple spouses or partners with whom you are considering childbearing, please remember that these questions pertain to the partner who was screened for study eligibility. If P3 is not equal to 1 then skip to instruction before C1If not, then skip to C1.The next few questions are related to your relationship with your partner with whom you are considering having a child, or with whom you have decided to delay or prevent pregnancy with.C1.	What is the nature of your relationship with this partner?  (Choose one)	1	In a committed relationship	2	Married	3	Friend	4	Other	8	Refuse to AnswerC2.	How old is your partner/spouse?	__ __	Years	98	Refuse to AnswerC3.	How long have you been together with your partner/ spouse?	__ __	YEARS	__ __	MONTHS	98	Refuse to Answer (Years)C4.	Do you live with this partner?	1	Yes	0	No	8	Refuse to AnswerC5a.	Do you have other spouses or partners with whom you are in a committed relationship?	1	Yes	0	No	8	Refuse to AnswerC5b.	Does your partner have other spouses or partners with whom they are in a committed relationship?  (Choose one)	0	No	1	Yes	7	Not Sure	8	Refuse to AnswerIf P3 is equal to 1, then skip to C6b.C6a.	Does your partner know you are HIV+?	1	Yes	0	No	7	Don't Know	8	Refuse to AnswerIf C6a is equal to 0 or C6a is equal to "Don't Know" or C6a is equal to "Refuse to Answer", then skip to C7a.C6b.	Did your partner learn of your HIV status from you?	1	Yes	0	No	8	Refuse to AnswerC6c.	How did your partner respond to finding out you are HIV+?  (Choose one)	1	My partner had a positive or supportive reaction	2	My partner had a negative or unsupportive reaction	3	My partner did not have much of a reaction; neither negative nor positive	8	Refuse to AnswerIf P3 is equal to 1, then skip to instruction before D1.C7a.	What is the HIV status of your partner?  (Choose one)	1	HIV-negative	2	HIV-positive	3	Unknown	8	Refuse to AnswerC7b.	When was your partner last tested for HIV?  (Choose one)	1	Within last 6 months	2	6-12 months	3	More than 12 months	4	Never tested	7	Don't Know	8	Refuse to AnswerIf (C7a is equal to 1 or C7a is equal to 3) and B6 is equal to 1 and (B6a is equal to 0 or B6a is equal to "Refuse to Answer"), then skip to instruction before E1a.If (C7a is equal to 1 or C7a is equal to 3) and (B6 is equal to 2 or B6a is equal to 1), then skip to instruction before E1a.C7c.	Is this partner taking ARVs to manage their HIV?	1	Yes	0	No	7	Don't Know	8	Refuse to AnswerIf B6 is equal to 1 and (B6a is equal to 0 or B6a is equal to "Refuse to Answer"), then skip to instruction before E1a.If B6 is equal to 2 or B6a is equal to 1, then skip to instruction before E1a.Reproductive historyThe following questions ask about pregnancy history and number of children.D1.	[D1_F]	__ __	Pregnancies	98	Refuse to AnswerIf D1 is equal to 0 or D1 is equal to "Refuse to Answer", then skip to D3.D1a.	Have you or your partner had any pregnancies in the 2 years prior to enrolling in the study?  (Choose one)	0	No, neither of us did, to the best of my knowledge	1	Yes, I did, with my current partner	2	Yes, I did, but with another partner	3	Yes, my partner did, but with someone else	7	Don't Know	8	Refuse to AnswerD2a.	How many of these pregnancies resulted in live births?	__ __	Pregnancies	97	Don't Know	98	Refuse to AnswerD2b.	How many resulted in a miscarriage or spontaneous abortion?	__ __	Pregnancies	97	Don't Know	98	Refuse to AnswerD2c.	How many resulted in an induced/planned abortion?	__ __	Pregnancies	97	Don't Know	98	Refuse to AnswerD2d.	How many resulted in a stillbirth?	__ __	Pregnancies	97	Don't Know	98	Refuse to AnswerD3.	Have you/your partner ever had difficulty conceiving a child, meaning you tried to get pregnant over an extended period of time but were unsuccessful?	1	Yes	0	No	7	Don't Know	8	Refuse to AnswerD4a.	How many children have you had? Please consider your own biological children, as well as children for whom you were not the biological parent.  iological children:	__ __	Biological children	98	Refuse to AnswerD4b.	How many children have you had?   on-biological children:	__ __	Non-biological children	98	Refuse to AnswerD4c.	How many biological and non-biological children does your partner have that are NOT included among your children?  iological children:	__ __	Biological children	97	Don't Know	98	Refuse to AnswerD4d.	Non-biological children:	__ __	Non-biological children	97	Don't Know	98	Refuse to AnswerIf (D1 is equal to 0 or D1 is equal to "Refuse to Answer" or D4a is equal to 0 or D4a is equal to "Refuse to Answer") and (P3 is equal to 1 or B6 is equal to 1 and (B6a is equal to 0 or B6a is equal to "Refuse to Answer")), then skip to instruction before E1a.If (D1 is equal to 0 or D1 is equal to "Refuse to Answer" or D4a is equal to 0 or D4a is equal to "Refuse to Answer") and P3 is greater than 1 and (B6 is equal to 0 or B6 is equal to 2 or B6 is equal to "Refuse to Answer"), then skip to instruction before G1.D5.	How many biological children have you had since you've known you were HIV+?	__ __	Biological children	98	Refuse to AnswerIf D5 is greater than D4a then The number of children should not be greater than number of your biological children (&[D4a]). and skip to D5.D6.	How many of your biological children were born HIV+?	__ __	Biological children	98	Refuse to AnswerIf D6 is greater than D4a then The number of children should not be greater than number of your biological children (&[D4a]). and skip to D6.D7.	How many biological children have you had with your current partner?	__ __	Biological children	98	Refuse to AnswerIf D7 is greater than D4a then The number of children should not be greater than number of your biological children (&[D4a]). and skip to D7.If P3 is greater than 1 and (B6 is equal to 0 or B6 is equal to 2 or B6 is equal to "Refuse to Answer"), then skip to instruction before G1.Intimate Partner ViolenceDHS survey:  Kishor, S., & Johnson, K. (2004). Profiling domestic violence: A multi-country study. Calverton, MD: ORC MacroIf E2 is equal to 0 and E3 is equal to 0 and E4 is equal to 0 and E6 is equal to 0 and E7 is equal to 0 and E8 is equal to 0 and (B6 is equal to 2 or B6 is equal to 8 or P3 is equal to 1), then skip to instruction before F1a.If E2 is equal to 0 and E3 is equal to 0 and E4 is equal to 0 and E6 is equal to 0 and E7 is equal to 0 and E8 is equal to 0 and (B6 is equal to 0 or B6 is equal to 1), then skip to instruction before G1.If E2 is equal to 0 and E3 is equal to 0 and E4 is equal to 0, then skip to E10a.E9a.	Has your partner engaged in these physical acts because the two of you disagreed about trying to have a child?  (Choose one)	0	No, this was not a factor	1	Yes it was one factor, but there were other reasons as well	2	Yes it was the main reason	5	I don't know whether this was a reason	8	Refuse to AnswerE9b.	Has your partner engaged in these physical acts because you and your partner were having a hard time conceiving a child?  (Choose one)	0	No, this was not a factor	1	Yes it was one factor, but there were other reasons as well	2	Yes it was the main reason	5	I don't know whether this was a reason	8	Refuse to AnswerIf E9a is not equal to 1 and E9a is not equal to 2 and E9b is not equal to 1 and E9b is not equal to 2 and E6 is equal to 0 and E7 is equal to 0 and E8 is equal to 0 and (B6 is equal to 2 or B6 is equal to 8 or P3 is equal to 1), then skip to instruction before F1a.If E9a is not equal to 1 and E9a is not equal to 2 and E9b is not equal to 1 and E9b is not equal to 2 and E6 is equal to 0 and E7 is equal to 0 and E8 is equal to 0 and (B6 is equal to 0 or B6 is equal to 1), then skip to instruction before G1.If E9a is not equal to 1 and E9a is not equal to 2 and E9b is not equal to 1 and E9b is not equal to 2, then skip to E10a.E9c.	Can you tell me a bit about this? When did these physical acts start? Did these physical acts start once you got in the study or met with the providers to learn about safer conception?	__ __ __ __ __ __ __ __ __ __ __ __ __ __ __ __ __ __ __ __ __ __ __ __ __ __ __ __ __ __ __ __ __ __ __ __ __ __ __ __ __ __ __ __ __ __ __ __ __ __ __ __ __ __ __ __ __ __ __ __ __ __ __ __ __ __ __ __ __ __ __ __ __ __ __ __ __ __ __ __ __ __ __ __ __ __ __ __ __ __ __ __ __ __ __ __ __ __ __ __ __ __ __ __ __ __ __ __ __ __ __ __ __ __ __ __ __ __ __ __ __ __ __ __ __ __ __ __ __ __ __ __ __ __ __ __ __ __ __ __ __ __ __ __ __ __ __ __ __ __ __ __ __ __ __ __ __ __ __ __ __ __ __ __ __ __ __ __ __ __ __ __ __ __ __ __ __ __ __ __ __ __ __ __ __ __ __ __ __ __ __ __ __ __ __ __ __ __ __ __If E6 is equal to 0 and E7 is equal to 0 and E8 is equal to 0 and (B6 is equal to 2 or B6 is equal to 8 or P3 is equal to 1), then skip to instruction before F1a.If E6 is equal to 0 and E7 is equal to 0 and E8 is equal to 0 and (B6 is equal to 0 or B6 is equal to 1), then skip to instruction before G1.E10a.	Have you engaged in these physical acts because the two of you disagreed about trying to have a child?  (Choose one)	0	No, this was not a factor	1	Yes it was one factor, but there were other reasons as well	2	Yes it was the main reason	5	I don't know whether this was a reason	8	Refuse to AnswerE10b.	Have you engaged in these physical acts because you and your partner were having a hard time conceiving a child?  (Choose one)	0	No, this was not a factor	1	Yes it was one factor, but there were other reasons as well	2	Yes it was the main reason	5	I don't know whether this was a reason	8	Refuse to AnswerIf E10a is not equal to 1 and E10a is not equal to 2 and E10b is not equal to 1 and E10b is not equal to 2 and (B6 is equal to 2 or B6 is equal to 8 or P3 is equal to 1), then skip to instruction before F1a.If E10a is not equal to 1 and E10a is not equal to 2 and E10b is not equal to 1 and E10b is not equal to 2 and (B6 is equal to 0 or B6 is equal to 1), then skip to instruction before G1.E10c.	Can you tell me a bit about this? When did these physical acts start? Did these physical acts start once you got in the study or met with the providers to learn about safer conception?	__ __ __ __ __ __ __ __ __ __ __ __ __ __ __ __ __ __ __ __ __ __ __ __ __ __ __ __ __ __ __ __ __ __ __ __ __ __ __ __ __ __ __ __ __ __ __ __ __ __ __ __ __ __ __ __ __ __ __ __ __ __ __ __ __ __ __ __ __ __ __ __ __ __ __ __ __ __ __ __ __ __ __ __ __ __ __ __ __ __ __ __ __ __ __ __ __ __ __ __ __ __ __ __ __ __ __ __ __ __ __ __ __ __ __ __ __ __ __ __ __ __ __ __ __ __ __ __ __ __ __ __ __ __ __ __ __ __ __ __ __ __ __ __ __ __ __ __ __ __ __ __ __ __ __ __ __ __ __ __ __ __ __ __ __ __ __ __ __ __ __ __ __ __ __ __ __ __ __ __ __ __ __ __ __ __ __ __ __ __ __ __ __ __ __ __ __ __ __ __If B6 is equal to 0 or B6 is equal to 1, then skip to instruction before G1.Sexual Relationship Power ScalePulerwitz J, Gortmaker SL, & DeJong W. Measuring Relationship Power in HIV/STD Research. Sex Roles. Vol. 42, April 2000.DepressionCox JL, Holden JM, Sagovsky R. Detection of postnatal depression. Development of the 10-item Edinburgh Postnatal Depression Scale. Br J Psychiatry. 1987;150:782–786Stigma measuresKalichman SC, Simbayi LC, Jooste S, Toefy Y, Cain D, Cherry C, Kagee A. Development of a brief scale to measure AIDS-related stigma in South Africa. AIDS Behav 2005;9(2):135-143.Perceived community childbearing stigmaThe next several questions are about your attitudes and perceptions of how others think about people living with HIV having children.H2a.	People in the community look down on HIV+ individuals who want to have a child.  (Choose one)	1	Disagree Strongly	2	Disagree Slightly	3	Neutral	4	Agree Slightly	5	Agree Strongly	8	Refuse to AnswerH2b.	An HIV+ man who gets his partner pregnant is looked down upon.  (Choose one)	1	Disagree Strongly	2	Disagree Slightly	3	Neutral	4	Agree Slightly	5	Agree Strongly	8	Refuse to AnswerH2c.	An HIV+ woman who gets pregnant is looked down upon.  (Choose one)	1	Disagree Strongly	2	Disagree Slightly	3	Neutral	4	Agree Slightly	5	Agree Strongly	8	Refuse to AnswerPerceived provider childbearing stigmaH3a.	Most HIV providers think that HIV+ clients should not have children.  (Choose one)	1	Disagree Strongly	2	Disagree Slightly	3	Neutral	4	Agree Slightly	5	Agree Strongly	8	Refuse to AnswerH3b.	Most HIV providers would support HIV+ clients who wish to have children.  (Choose one)	1	Disagree Strongly	2	Disagree Slightly	3	Neutral	4	Agree Slightly	5	Agree Strongly	8	Refuse to AnswerAchievement of desired pregnancy statusAnderson JC, Grace KT, Miller E. Reproductive coercion among women living with HIV: an unexplored risk factor for negative sexual and mental health outcomes. AIDS 2017; 31:2261-5If P3 is equal to 1, then skip to instruction before I2a.I1.	Have you/your partner had any pregnancies since your last study interview?  (Choose one)	0	No	1	Yes	2	Same pregnancy as reported during last interview	8	Refuse to AnswerIf I1 is equal to 1, then skip to instruction before I8a.If I1 is equal to 2, then skip to I9.I2a.	Do you want a (another) child?  (Choose one)	1	Yes, now	2	Yes, but later	3	Not at all	7	Don't Know	8	Refuse to AnswerI2b.	Does your partner want a (another) child?  (Choose one)	1	Yes, now	2	Yes, but later	3	Not at all	7	Don't Know	8	Refuse to AnswerInternalized childbearing stigmaTell me the extent to which you agree or disagree with the following statement.I3.	[I3_F]  (Choose one)	1	Disagree Strongly	2	Disagree Slightly	3	Neutral	4	Agree Slightly	5	Agree Strongly	8	Refuse to AnswerI4.	Do you and your partner intend or have plans to become pregnant?  (Choose one)	0	No, not at all	1	Yes, we are trying now to get pregnant	2	Yes, within 6-12 months from now	3	Yes, but more than 1 year from now	7	Don't Know	8	Refuse to AnswerIf I4 is not equal to 1, then skip to I7.I5.	For how long have you and your partner been trying to have a(nother) child?  (Choose one)	0	0-6 months	1	7-12 months	2	3-24 months	3	For more than 2 years	7	Don't Know	8	Refuse to AnswerI6a.	Have you or your partner had any tests done to assess for infertility (inability to have children)?	1	Yes	0	No	8	Refuse to AnswerI6b.	Have you or your partner been told by a provider that you may have problems with infertility?	1	Yes	0	No	8	Refuse to AnswerIf I4 is equal to 1, then skip to instruction before K1a.I7.	Tell me why you are not currently trying to get pregnant. (Interviewer: Do not read the following; just use these to code the response)  (Check all that apply)  (Check all that apply)	__	No longer in a relationship (self/partner ended the relationship; partner died)	__	The discussion with our counselor/provider led us to this decision	__	I or my partner no longer wants or is willing to have a child	__	Had a pregnancy since last survey and had a miscarriage, abortion or a still birth	__	Had a pregnancy since last survey, and had a live birth or is still pregnant	__	My or my partner's health has declined significantly (poor health)	__	Have been told that I or my partner are infertile	__	Other	__	Refuse to AnswerIf I7B is not equal to 1, then skip to instruction before I7_c.I7_b.	What about the discussion led to you changing your mind?	__ __ __ __ __ __ __ __ __ __ __ __ __ __ __ __ __ __ __ __ __ __ __ __ __ __ __ __ __ __ __ __ __ __ __ __ __ __ __ __ __ __ __ __ __ __ __ __ __ __ __ __ __ __ __ __ __ __ __ __ __ __ __ __ __ __ __ __ __ __ __ __ __ __ __ __ __ __ __ __ __ __ __ __ __ __ __ __ __ __ __ __ __ __ __ __ __ __ __ __If I7C is not equal to 1, then skip to instruction before I7_s.I7_c.	What led you to change your mind?	__ __ __ __ __ __ __ __ __ __ __ __ __ __ __ __ __ __ __ __ __ __ __ __ __ __ __ __ __ __ __ __ __ __ __ __ __ __ __ __ __ __ __ __ __ __ __ __ __ __ __ __ __ __ __ __ __ __ __ __ __ __ __ __ __ __ __ __ __ __ __ __ __ __ __ __ __ __ __ __ __ __ __ __ __ __ __ __ __ __ __ __ __ __ __ __ __ __ __ __If I7H is not equal to 1, then skip to instruction before I8a.I7_s.	Specify other reason you are not currently trying to get pregnant:	__ __ __ __ __ __ __ __ __ __ __ __ __ __ __ __ __ __ __ __ __ __ __ __ __ __ __ __ __ __ __ __ __ __ __ __ __ __ __ __ __ __ __ __ __ __ __ __ __ __ __ __ __ __ __ __ __ __ __ __ __ __ __ __ __ __ __ __ __ __ __ __ __ __ __ __ __ __ __ __ __ __ __ __ __ __ __ __ __ __ __ __ __ __ __ __ __ __ __ __If P3 is equal to 1 or P3 is greater than 1 and I1 is equal to 0, then skip to instruction before K1a.INTERVIEWER: For male respondents, if their female partner is available to respond to questions, she should answer I8a to J13; she should be asked alone, without the male participant in the room.I8a.	When did you learn of this pregnancy?	__ __ / __ __ / __ __ __ __	mm / dd / yyyy	2098	Refuse to Answer (Year)If TODAY - I8a is greater than 540 and I8a is not equal to "Refuse to Answer" then Date when learned of pregnancy cannot be greater than 18 months ago. and skip to I8a.I8b.	How did you determine that you (or your partner) were pregnant?  (Choose one)	1	Pregnancy test at home	2	Pregnancy test at health center	3	Assumed due to missed/late menstrual period	4	Other	7	Don't Know	8	Refuse to AnswerIf I8b is not equal to 4, then skip to I9.I8B_s.	Specify other way determined pregnancy:	__ __ __ __ __ __ __ __ __ __ __ __ __ __ __ __ __ __ __ __ __ __ __ __ __ __ __ __ __ __ __ __ __ __ __ __ __ __ __ __ __ __ __ __ __ __ __ __ __ __ __ __ __ __ __ __ __ __ __ __ __ __ __ __ __ __ __ __ __ __ __ __ __ __ __ __ __ __ __ __ __ __ __ __ __ __ __ __ __ __ __ __ __ __ __ __ __ __ __ __I9.	What is the current status of this pregnancy?  (Choose one)	1	Still pregnant	2	Miscarriage/ spontaneous abortion	3	Induced abortion	4	Still birth	5	Live birth	8	Refuse to AnswerIf I9 is equal to 4 or I9 is equal to 5, then skip to J1a.If I9 is equal to 2 or I9 is equal to 3, then skip to I11.I10.	When is the expected delivery date?	__ __ / __ __ / __ __ __ __	mm / dd / yyyy	2098	Refuse to Answer (Year)If I10 - TODAY is greater than 365 and I10 is not equal to "Refuse to Answer" then Expected delivery date cannot be more than 12 months in the future. and skip to I10.If I9 is equal to 1, then skip to instruction before K1a.I11.	Approximately how many weeks were you (or your partner) pregnant at the time that you learned of the miscarriage/abortion?	__ __	weeks	98	Refuse to AnswerI12.	Were you (was she) seen by a provider at that time?	1	Yes	0	No	7	Don't Know	8	Refuse to AnswerIf I9 is equal to 3, then skip to J1a.I13.	How did you feel about the miscarriage?  (Choose one)	1	I was very upset about it	2	I was somewhat upset about it	3	I was not that upset, because I was no longer sure whether I wanted the child	8	Refuse to AnswerI14.	What physical signs or symptoms indicated that you/she experienced a miscarriage?	__ __ __ __ __ __ __ __ __ __ __ __ __ __ __ __ __ __ __ __ __ __ __ __ __ __ __ __ __ __ __ __ __ __ __ __ __ __ __ __ __ __ __ __ __ __ __ __ __ __ __ __ __ __ __ __ __ __ __ __ __ __ __ __ __ __ __ __ __ __ __ __ __ __ __ __ __ __ __ __ __ __ __ __ __ __ __ __ __ __ __ __ __ __ __ __ __ __ __ __I15.	Do you have any thoughts as to what may have caused the miscarriage?	__ __ __ __ __ __ __ __ __ __ __ __ __ __ __ __ __ __ __ __ __ __ __ __ __ __ __ __ __ __ __ __ __ __ __ __ __ __ __ __ __ __ __ __ __ __ __ __ __ __ __ __ __ __ __ __ __ __ __ __ __ __ __ __ __ __ __ __ __ __ __ __ __ __ __ __ __ __ __ __ __ __ __ __ __ __ __ __ __ __ __ __ __ __ __ __ __ __ __ __PMTCT/pregnancy care processes adherenceJ1a.	Did you (your partner) receive antenatal care (ANC) from a clinic during the pregnancy?	1	Yes	0	No	8	Refuse to AnswerIf J1a is equal to 0 or J1a is equal to "Refuse to Answer", then skip to J2.J1b.	How many total ANC visits were attended?	__ __	98	Refuse to AnswerJ1c.	At what gestational age of pregnancy did you attend the first ANC visit? Interviewer: If respondent is uncertain, ask them to consider the # of weeks since missed menstrual period)	__ __	weeks	98	Refuse to AnswerJ1d.	Did you attend any antenatal care visits with your partner during the pregnancy?	1	Yes	0	No	8	Refuse to AnswerJ2.	Did you (your partner) use PMTCT medication or ARVs during pregnancy to prevent HIV transmission to the child?  (Choose one)	0	No	1	Yes, I/she was on ART prior to pregnancy	2	Yes, I/she started on ART during (after learning of) pregnancy	7	Don't Know	8	Refuse to AnswerIf J2 is equal to 1, then skip to J5.If J2 is equal to 2, then skip to J4.J3.	What was the reason(s) you (your partner) did not use ARVs?  (Check all that apply)  (Check all that apply)	__	Partner is not HIV+	__	ARVs not offered by providers	__	ARVs not available	__	I (my partner) am (is) still pregnant and may still start ARVs	__	Concern about side effects	__	Other	__	Don't Know	__	Refuse to AnswerIf (J2 is equal to 0 or J2 is equal to "Don't Know" or J2 is equal to "Refuse to Answer") and (I9 is equal to 4 or I9 is equal to 5), then skip to instruction before J7a.If (J2 is equal to 0 or J2 is equal to "Don't Know" or J2 is equal to "Refuse to Answer") and (I9 is equal to 2 or I9 is equal to 3), then skip to J13.If J2 is equal to 1, then skip to J5.J4.	At what gestational age of pregnancy did you (your partner) start on ARVs?	__ __	weeks	98	Refuse to AnswerJ5.	How many times did you (your partner) miss a dose of the ARVs (best guess) during pregnancy?  If treatment was discontinued, select "Not Applicable".	__ __ __	missed doses	998	Refuse to Answer	999	Not ApplicableIf (J5 is equal to 0 or J5 is equal to "Refuse to Answer" or J5 is equal to "Not Applicable") and (I9 is equal to 4 or I9 is equal to 5), then skip to instruction before J7a.If (J5 is equal to 0 or J5 is equal to "Refuse to Answer" or J5 is equal to "Not Applicable") and (I9 is equal to 2 or I9 is equal to 3), then skip to J13.J6.	What were the main reasons for missing (stopping) the ARVs?  (Check all that apply)  (Check all that apply)	__	Side effects	__	Ran out of drugs	__	No transport to the health facility	__	Forgot	__	Other reasons	__	Refuse to AnswerIf I9 is equal to 2 or I9 is equal to 3, then skip to J13.The next few questions relate to the time period since the child was delivered.J7a.	Where did you (your partner) deliver?  (Choose one)	1	Health facility	2	Home	3	On the way to the health facility	4	Location of traditional birth attendant	5	Other	8	Refuse to AnswerJ7b.	At what week of gestation was the infant delivered?	__ __	weeks	98	Refuse to AnswerJ7c.	What was the child's weight at birth?	__ __	Kg	98	Refuse to AnswerIf I9 is equal to 4, then skip to J13.J8a.	Was your child given nevirapine prophylaxis? (Interviewer: show the pill type)	1	Yes	0	No	7	Don't Know	8	Refuse to AnswerIf J8a is equal to 0 or J8a is equal to "Don't Know" or J8a is equal to "Refuse to Answer", then skip to J9.J8b.	When was the child started on nevirapine?  If right after birth, enter '0' days.	__ __ __ __	Days	9998	Refuse to AnswerJ9.	Was your child put on cotrimoxazole prophylaxis? (Interviewer: show the pill type)	1	Yes	0	No	7	Don't Know	8	Refuse to AnswerJ10a.	Has the child been tested for HIV?	1	Yes	0	No	7	Don't Know	8	Refuse to AnswerIf J10a is equal to 0 or J10a is equal to "Don't Know" or J10a is equal to "Refuse to Answer", then skip to J12.J10b.	How old was the baby when the HIV test was first done?	__ __ __	Weeks	998	Refuse to AnswerJ10c.	Please share with me the outcome of the child's latest HIV test  (Choose one)	1	Negative	2	Positive	3	Did not receive results	4	Indeterminant/test failed	8	Refuse to AnswerIf J10c is equal to 1 or J10c is equal to 3 or J10c is equal to 4, then skip to J12.J11.	Is the child on ARVs?	1	Yes	0	No	7	Don't Know	8	Refuse to AnswerJ12.	What are you giving the child to eat and drink? (check all that apply)  (Check all that apply)	__	Water	__	Breast milk	__	Replacement feeding (formula)	__	Food/porridge	__	Other	__	Refuse to AnswerJ13.	Do you and/or your partner want to try and have another child in the future?  (Choose one)	0	No, neither of us want another child	1	Yes, my partner does, but I don't	2	Yes, I do, but my partner does not	3	Yes, we both do	4	We are not sure	8	Refuse to AnswerINTERVIEWER: J14a-d should be asked of the index participant, even if female partner is available (for male participants)Partner PMTCT/Pregnancy SupportStapleton LRT, Schetter CD, Westling E, Rini C, Glynn LM, Hobel CJ, Sandman CA. Perceived partner support in pregnancy predicts lower maternal and infant distress. J Fam Psychol. 2012; 26:453-63.Contraception UseThe next series of questions relate to the use of contraception to prevent pregnancy. NTERVIEWER: For male respondents, if their female partner is available to respond to questions, she should answer all of section K (alone, without male partner in the room), EXCEPT for section related to male condom use.K1a.	Are (Is) you (your partner) currently using any contraceptive method to prevent pregnancy?	1	Yes	0	No	7	Don't Know	8	Refuse to AnswerK1b.	Have you (your partner) used emergency contraception (morning after pill) in the past 6 months?	1	Yes	0	No	7	Don't Know	8	Refuse to AnswerIf (K1b is equal to 0 or K1b is equal to "Don't Know" or K1b is equal to "Refuse to Answer") and (K1a is equal to 0 or K1a is equal to "Don't Know" or K1a is equal to "Refuse to Answer"), then skip to K191.If K1b is equal to 0 or K1b is equal to "Don't Know" or K1b is equal to "Refuse to Answer", then skip to K2a.K1c.	How many times in the past 6 months have you (your partner) used emergency contraceptive (morning after pill)?	__ __	times	98	Refuse to AnswerIf K1a is equal to 0 or K1a is equal to "Don't Know" or K1a is equal to "Refuse to Answer", then skip to K191.K2a.	What is your PRIMARY method of contraception? (select one)  (Choose one)	01	Injectables	02	Oral contraceptives (birth control pills)	03	Intrauterine device (IUD; the coil)	04	Implant (put in forearm)	05	Female sterilization (tying woman's tubes)	06	Male sterilization (tying men's tubes)	07	Condoms (for the male)	08	Condoms (for the female)	09	Traditional method [LAM, withdrawal, fertility based awareness (FAMS)/rhythm]	10	Other	98	Refuse to AnswerIf K2a is not equal to 10, then skip to K2b.K2a_s.	Specify other method of contraception:	__ __ __ __ __ __ __ __ __ __ __ __ __ __ __ __ __ __ __ __ __ __ __ __ __ __ __ __ __ __ __ __ __ __ __ __ __ __ __ __ __ __ __ __ __ __ __ __ __ __ __ __ __ __ __ __ __ __ __ __ __ __ __ __ __ __ __ __ __ __ __ __ __ __ __ __ __ __ __ __ __ __ __ __ __ __ __ __ __ __ __ __ __ __ __ __ __ __ __ __K2b.	How long ago did you (your partner) start using this method of contraception?	__ __ __	months	998	Refuse to AnswerK2c.	[K2C_F]  (Choose one)	0	&[K2C0_F]	1	&[K2C1_F]	2	&[K2C2_F]	3	&[K2C3_F]	8	Refuse to AnswerIf P6 is equal to 1 and K2c is equal to 0 then This choice is not valid for Male respondents. and skip to K2c.If P7 is equal to 0 and K2a is not equal to 7 and K2a is not equal to 6, then skip to K100.If K2a is equal to 1, then skip to K10.If K2a is equal to 2, then skip to K20.If K2a is equal to 3, then skip to K30a.If K2a is equal to 4, then skip to K40a.If K2a is equal to 5 or K2a is equal to 6, then skip to K50.If K2a is equal to 7 or K2a is equal to 8, then skip to K60.If K2a is equal to 9, then skip to K70.If K2a is equal to 10, then skip to K100.Injectable contraceptivesK10.	With what frequency are the injections recommended?  (Choose one)	1	Every month	2	Every 2 months	3	Every 3 months	4	Every 6 months	8	Refuse to AnswerK11.	How would you describe the timeliness of your last injection?  (Choose one)	1	I received the injection on time	2	I received the injection less than 1 week late	3	I received the injection between 1 and 2 weeks late	4	I received the injection between 2-4 weeks late	5	I received the injection greater than 4 weeks late	6	I did not receive the injection	8	Refuse to AnswerK12a.	In the past 6 months, have you returned to the clinic to receive your contraceptive injection and found that it was unavailable?	1	Yes	0	No	8	Refuse to AnswerIf K12a is equal to 0 or K12a is equal to "Refuse to Answer", then skip to K13.K12b.	What advice did you receive from your provider?  (Choose one)	1	I was referred to another facility to receive my injection	2	I was advised to use condoms	3	I was advised to switch to another method of contraception	4	I was given a date to return to the hospital	5	Other	8	Refuse to AnswerIf K12b is not equal to 5, then skip to K13.K12b_s.	Explain other advice:	__ __ __ __ __ __ __ __ __ __ __ __ __ __ __ __ __ __ __ __ __ __ __ __ __ __ __ __ __ __ __ __ __ __ __ __ __ __ __ __ __ __ __ __ __ __ __ __ __ __ __ __ __ __ __ __ __ __ __ __ __ __ __ __ __ __ __ __ __ __ __ __ __ __ __ __ __ __ __ __ __ __ __ __ __ __ __ __ __ __ __ __ __ __ __ __ __ __ __ __K13.	Have you experienced any side effects from the use of this contraceptive in the past month? If so, how severe were the side effects?  (Choose one)	0	No side effects	1	Minor side effects	2	Moderate side effects	3	Severe side effects	8	Refuse to AnswerPlease indicate the extent to which you agree with each of the following statements.K14a.	I don't want to get pregnant again right now.  (Choose one)	1	Strongly Agree	2	Agree	3	Disagree	4	Strongly Disagree	8	Refuse to AnswerK14b.	It is OK if I miss an injection.  (Choose one)	1	Strongly Agree	2	Agree	3	Disagree	4	Strongly Disagree	8	Refuse to AnswerK14c.	I worry that the injections affect my ability to breastfeed my infant.  (Choose one)	1	Strongly Agree	2	Agree	3	Disagree	4	Strongly Disagree	8	Refuse to AnswerK14d.	I find it difficult to return to the clinic for my injection.  (Choose one)	1	Strongly Agree	2	Agree	3	Disagree	4	Strongly Disagree	8	Refuse to AnswerK14e.	I am confident I will have enough money to continue my contraception.  (Choose one)	1	Strongly Agree	2	Agree	3	Disagree	4	Strongly Disagree	8	Refuse to AnswerK14f.	I worry my contraception may interact with other medications that I am on.  (Choose one)	1	Strongly Agree	2	Agree	3	Disagree	4	Strongly Disagree	8	Refuse to AnswerK14g.	I worry people will find out that I am using contraception injections.  (Choose one)	1	Strongly Agree	2	Agree	3	Disagree	4	Strongly Disagree	8	Refuse to AnswerK14h.	Due to my religious beliefs, I sometimes feel guilty that I am using injectable contraception.  (Choose one)	1	Strongly Agree	2	Agree	3	Disagree	4	Strongly Disagree	8	Refuse to AnswerSkip to K100.Oral ContraceptivesK20.	In the past month, approximately how many days have you missed taking your oral contraceptive?	__ __	days	98	Refuse to AnswerIf K20 is equal to 0 or K20 is equal to "Refuse to Answer", then skip to K22.K21.	Please select the reason(s) why you missed taking the contraceptive pill (select all that apply):  (Check all that apply)	__	I forgot	__	I did not want to take it in front of other people	__	I did not have my oral contraceptives with me	__	I was not feeling well	__	Side effects were too severe	__	Other	__	Refuse to AnswerIf K21F is not equal to 1, then skip to K22.K21f_s.	Specify other reason(s) why missed taking contraceptive pill	__ __ __ __ __ __ __ __ __ __ __ __ __ __ __ __ __ __ __ __ __ __ __ __ __ __ __ __ __ __ __ __ __ __ __ __ __ __ __ __ __ __ __ __ __ __ __ __ __ __ __ __ __ __ __ __ __ __ __ __ __ __ __ __ __ __ __ __ __ __ __ __ __ __ __ __ __ __ __ __ __ __ __ __ __ __ __ __ __ __ __ __ __ __ __ __ __ __ __ __K22.	In the past 6 months, have you run out of your oral contraceptive before you've been able to return to the clinic to refill your prescription.	1	Yes	0	No	8	Refuse to AnswerK23a.	In the past 6 months, have you returned to the clinic to pick up your oral contraceptive prescription and found that it was unavailable?	1	Yes	0	No	8	Refuse to AnswerIf K23a is equal to 0 or K23a is equal to "Refuse to Answer", then skip to K24.K23b.	How many times has this occurred?	__ __	98	Refuse to AnswerK23c.	What advice did you receive from your provider?  (Choose one)	1	I was referred to another facility to fill my prescription	2	I was advised to use condoms	3	I was advised to switch to another method of contraception	4	I was given a date to return to the hospital	5	Other	8	Refuse to AnswerIf K23c is not equal to 5, then skip to K24.K23c_s.	Specify other advice from provider:	__ __ __ __ __ __ __ __ __ __ __ __ __ __ __ __ __ __ __ __ __ __ __ __ __ __ __ __ __ __ __ __ __ __ __ __ __ __ __ __ __ __ __ __ __ __ __ __ __ __ __ __ __ __ __ __ __ __ __ __ __ __ __ __ __ __ __ __ __ __ __ __ __ __ __ __ __ __ __ __ __ __ __ __ __ __ __ __ __ __ __ __ __ __ __ __ __ __ __ __K24.	Have you experienced any side effects from the use of this contraceptive in the past month? If so, how severe were the side effects?  (Choose one)	0	No side effects	1	Minor side effects	2	Moderate side effects	3	Severe side effects	8	Refuse to AnswerPlease indicate what is the best action to be taken in each of the following scenarios.K25a.	You miss one or two days of your pills or start the pack one or two days late:  (Choose one)	1	Discard the missed pill(s) and then continue taking normally the next day	2	Take the missed pill(s) as soon as possible then continue taking normally	3	Throw away the pack and start a new pack after 5 days	4	Take the remaining pills in the pack and start a new pack the next day, with no break	8	Refuse to AnswerK25b.	You miss three or more days in the third week of pills:  (Choose one)	1	Discard the missed pill(s) and then continue taking normally the next day	2	Take the missed pill(s) as soon as possible then continue taking normally	3	Throw away the pack and start a new pack after 5 days	4	Take the remaining pills in the pack and start a new pack the next day, with no break	8	Refuse to AnswerPlease indicate the extent to which you agree with each of the following statements.K26a.	I don't want to get pregnant again right now.  (Choose one)	1	Strongly Agree	2	Agree	3	Disagree	4	Strongly Disagree	8	Refuse to AnswerK26b.	It is difficult to remember to take my pill every day.  (Choose one)	1	Strongly Agree	2	Agree	3	Disagree	4	Strongly Disagree	8	Refuse to AnswerK26c.	It is OK if I forget to take my pills for three days in a row.  (Choose one)	1	Strongly Agree	2	Agree	3	Disagree	4	Strongly Disagree	8	Refuse to AnswerK26d.	I worry that my oral contraceptives affect my ability to breastfeed my infant.  (Choose one)	1	Strongly Agree	2	Agree	3	Disagree	4	Strongly Disagree	8	Refuse to AnswerK26e.	I find it difficult to return to the clinic to refill my prescription in time.  (Choose one)	1	Strongly Agree	2	Agree	3	Disagree	4	Strongly Disagree	8	Refuse to AnswerK26f.	I am confident I will have enough money to continue my contraception.  (Choose one)	1	Strongly Agree	2	Agree	3	Disagree	4	Strongly Disagree	8	Refuse to AnswerK26g.	I worry my contraception may interact with other medications that I am on.  (Choose one)	1	Strongly Agree	2	Agree	3	Disagree	4	Strongly Disagree	8	Refuse to AnswerK26h.	I worry people will find out that I am using contraception pills.  (Choose one)	1	Strongly Agree	2	Agree	3	Disagree	4	Strongly Disagree	8	Refuse to AnswerK26i.	Due to my religious beliefs, I sometimes feel guilty that I am using contraception pills.  (Choose one)	1	Strongly Agree	2	Agree	3	Disagree	4	Strongly Disagree	8	Refuse to AnswerSkip to K100.Intrauterine Device (IUD)K30a.	Approximately how long ago was your IUD inserted:	__ __ __	YEARS	__ __ __	MONTHS	998	Refuse to Answer (Months)K30b.	In approximately how many years should your IUD be removed and replaced?	__ __	years	98	Refuse to AnswerK31.	Have you experienced any side effects from the use of this contraceptive in the past month? If so, how severe were the side effects?  (Choose one)	0	No side effects	1	Minor side effects	2	Moderate side effects	3	Severe side effects	8	Refuse to AnswerIndicate whether each of the following is true or false.K32a.	Women who have not had children cannot use IUD.  (Choose one)	0	False	1	True	7	Don't Know	8	Refuse to AnswerK32b.	An IUD can protect a woman from pregnancy for 12-13 years.  (Choose one)	0	False	1	True	7	Don't Know	8	Refuse to AnswerK32c.	An IUD can migrate to other parts of the body and cause infection.  (Choose one)	0	False	1	True	7	Don't Know	8	Refuse to AnswerK32d.	Women who are breastfeeding can use IUD.  (Choose one)	0	False	1	True	7	Don't Know	8	Refuse to AnswerK32e.	Fertility returns immediately after removal of IUD.  (Choose one)	0	False	1	True	7	Don't Know	8	Refuse to AnswerK32f.	An IUD causes pain and discomfort during intercourse.  (Choose one)	0	False	1	True	7	Don't Know	8	Refuse to AnswerPlease indicate the extent to which you agree with each of the following statements.K33a.	I don't want to get pregnant again right now.  (Choose one)	1	Strongly Agree	2	Agree	3	Disagree	4	Strongly Disagree	8	Refuse to AnswerK33b.	I am confident I will have enough money to continue my contraception.  (Choose one)	1	Strongly Agree	2	Agree	3	Disagree	4	Strongly Disagree	8	Refuse to AnswerK33c.	It will be difficult to continue to use this contraceptive over time.  (Choose one)	1	Strongly Agree	2	Agree	3	Disagree	4	Strongly Disagree	8	Refuse to AnswerK33d.	I worry that my contraception may interact with other medications I am on.  (Choose one)	1	Strongly Agree	2	Agree	3	Disagree	4	Strongly Disagree	8	Refuse to AnswerK33e.	I worry people will find out I am using an IUD.  (Choose one)	1	Strongly Agree	2	Agree	3	Disagree	4	Strongly Disagree	8	Refuse to AnswerK33f.	Due to my religious beliefs, I sometimes feel guilty that I am using an IUD.  (Choose one)	1	Strongly Agree	2	Agree	3	Disagree	4	Strongly Disagree	8	Refuse to AnswerSkip to K100.ImplantsK40a.	Approximately how long ago was your implant inserted:	__ __ __	YEARS	__ __ __	MONTHS	998	Refuse to Answer (Months)K40b.	In approximately how many years or months should your implant be removed and replaced?	__ __ __	YEARS	__ __ __	MONTHS	998	Refuse to Answer (Months)K41.	Have you experienced any side effects from the use of this contraceptive in the past month? If so, how severe were the side effects?  (Choose one)	0	No side effects	1	Minor side effects	2	Moderate side effects	3	Severe side effects	8	Refuse to AnswerIndicate whether each of the following is true or false.K42a.	Women who have not had children cannot use implants.  (Choose one)	0	False	1	True	7	Don't Know	8	Refuse to AnswerK42b.	An implant can protect a woman from pregnancy for 3-5 years.  (Choose one)	0	False	1	True	7	Don't Know	8	Refuse to AnswerK42c.	An implant can migrate to other parts of the body and cause infection.  (Choose one)	0	False	1	True	7	Don't Know	8	Refuse to AnswerK42d.	Women who are breastfeeding can use implants.  (Choose one)	0	False	1	True	7	Don't Know	8	Refuse to AnswerK42e.	Fertility returns after removal of the implant.  (Choose one)	0	False	1	True	7	Don't Know	8	Refuse to AnswerK42f.	Implants cause pain and discomfort during intercourse.  (Choose one)	0	False	1	True	7	Don't Know	8	Refuse to AnswerPlease indicate the extent to which you agree with each of the following statements.K43a.	I don't want to get pregnant again right now.  (Choose one)	1	Strongly Agree	2	Agree	3	Disagree	4	Strongly Disagree	8	Refuse to AnswerK43b.	I worry my contraceptive implant affects my ability to breastfeed my infant.  (Choose one)	1	Strongly Agree	2	Agree	3	Disagree	4	Strongly Disagree	8	Refuse to AnswerK43c.	I am confident I will have enough money to continue my contraception.  (Choose one)	1	Strongly Agree	2	Agree	3	Disagree	4	Strongly Disagree	8	Refuse to AnswerK43d.	It will be difficult to continue to use this contraceptive over time.  (Choose one)	1	Strongly Agree	2	Agree	3	Disagree	4	Strongly Disagree	8	Refuse to AnswerK43e.	I worry that my contraception may interact with other medications I am on.  (Choose one)	1	Strongly Agree	2	Agree	3	Disagree	4	Strongly Disagree	8	Refuse to AnswerK43f.	I worry people will find out I am using a contraception implant.  (Choose one)	1	Strongly Agree	2	Agree	3	Disagree	4	Strongly Disagree	8	Refuse to AnswerK43g.	Due to my religious beliefs, I sometimes feel guilty that I am using a contraception implant.  (Choose one)	1	Strongly Agree	2	Agree	3	Disagree	4	Strongly Disagree	8	Refuse to AnswerSkip to K100.SterilizationK50.	Have you experienced any side effects from the use of this contraceptive in the past month? If so, how severe were the side effects?  (Choose one)	0	No side effects	1	Minor side effects	2	Moderate side effects	3	Severe side effects	8	Refuse to AnswerIndicate whether each of the following is true or false.K51a.	Sterilization can be reversed when the couple decides to have another child.  (Choose one)	0	False	1	True	7	Don't Know	8	Refuse to AnswerK51b.	Sterilization can only be performed on women or men with 4 or more living children.  (Choose one)	0	False	1	True	7	Don't Know	8	Refuse to AnswerPlease indicate the extent to which you agree with each of the following statements.K52a.	I don't want (my partner) to get pregnant again right now.  (Choose one)	1	Strongly Agree	2	Agree	3	Disagree	4	Strongly Disagree	8	Refuse to AnswerK52b.	I worry that people will find out about the sterilization procedure.  (Choose one)	1	Strongly Agree	2	Agree	3	Disagree	4	Strongly Disagree	8	Refuse to AnswerIf K2a is equal to 6, then skip to K52d.K52c.	I worry that the sterilization affects my ability to breastfeed my infant.  (Choose one)	1	Strongly Agree	2	Agree	3	Disagree	4	Strongly Disagree	8	Refuse to AnswerK52d.	It is difficult to return to the clinic for follow up care from the sterilization.  (Choose one)	1	Strongly Agree	2	Agree	3	Disagree	4	Strongly Disagree	8	Refuse to AnswerK52e.	Due to my religious beliefs, I sometimes feel guilty about the sterilization.  (Choose one)	1	Strongly Agree	2	Agree	3	Disagree	4	Strongly Disagree	8	Refuse to AnswerSkip to K100.CondomsSunmola, Adegbenga M. "Developing a scale for measuring the barriers to condom use in Nigeria." Bulletin of the World Health Organization 79.10 (2001): 926-932.Skip to K100.Traditional MethodsK70.	Which of the traditional methods were used? (check all that apply)  (Check all that apply)	__	LAM	__	Withdrawal	__	Fertility awareness menstruations strategies (FAMS/rhythm)If K70A is equal to 1, then skip to instruction before K71a.If K70A is equal to 0 and K70C is equal to 1, then skip to K73.If K70A is equal to 0 and K70C is equal to 0 and K70B is equal to 1, then skip to K76.Indicate whether each of the following is true or false.K71a.	My menstrual cycle has not resumed since the birth of my last baby.  (Choose one)	0	False	1	True	7	Don't Know	8	Refuse to AnswerK71b.	I have started feeding my infant other foods and drinks, other than breastmilk.  (Choose one)	0	False	1	True	7	Don't Know	8	Refuse to AnswerK71c.	My baby is not yet 6 months.  (Choose one)	0	False	1	True	7	Don't Know	8	Refuse to AnswerPlease indicate whether LAM is an effective method of contraception in each of the following situations.K72a.	Your infant is less than 6 months of age and on replacement feeding.  (Choose one)	0	Not Effective	1	Effective	7	Don't Know	8	Refuse to AnswerK72b.	Your menses have returned and your infant is exclusively breastfeeding.  (Choose one)	0	Not Effective	1	Effective	7	Don't Know	8	Refuse to AnswerK72c.	You have not started menstruating and your infant is less than 6 months and exclusively breastfeeding.  (Choose one)	0	Not Effective	1	Effective	7	Don't Know	8	Refuse to AnswerK72d.	Your infant is 9 months of age, breastfeeding and starting on solid foods, and your menses have not yet returned.  (Choose one)	0	Not Effective	1	Effective	7	Don't Know	8	Refuse to AnswerIf K70C is equal to 1, then skip to K73.If K70B is equal to 0 and K70C is equal to 0, then skip to instruction before K78a.If K70C is equal to 0 and K70B is equal to 1, then skip to K76.K73.	Please indicate which of the following best describes your menstrual cycle.  (Choose one)	1	My cycle (time between bleeding) is usually less than 26 days	2	My cycle (time between bleeding) is usually between 26-32 days	3	My cycle (time between bleeding) is usually more than 32 days	4	I don't know how long my menstrual cycle usually is	8	Refuse to AnswerK74.	When in her menstrual cycle is a woman most fertile?  (Choose one)	1	When she is menstruating	2	1-7 days after bleeding begins	3	8-19 days after bleeding begins	4	15-21 days after bleeding begins	7	Don't Know	8	Refuse to AnswerK75.	How many fertile days per menstrual cycle must couples avoid unprotected intercourse or abstain from sex to prevent pregnancy while using FAMS?  (Choose one)	1	<5 days	2	8 days	3	10 days	4	12 days	5	14 days	7	Don't Know	8	Refuse to AnswerIf K70B is equal to 0, then skip to instruction before K78a.K76.	In the past 6 months, when having intercourse with your partner, how often have you (your partner) withdrawn before ejaculation?  (Choose one)	0	Never	1	Rarely	2	About half the time	3	Most of the time	4	Always	8	Refuse to AnswerIndicate whether each of the following is true or false.K77.	It is possible for a woman to get pregnant, even if her partner withdraws before ejaculation.  (Choose one)	0	False	1	True	7	Don't Know	8	Refuse to AnswerPlease indicate the extent to which you agree with each of the following statements.K78a.	I don't want to get pregnant again right now  (Choose one)	1	Strongly Agree	2	Agree	3	Disagree	4	Strongly Disagree	7	Don't Know	8	Refuse to AnswerK78b.	I find use of traditional methods disruptive to sex.  (Choose one)	1	Strongly Agree	2	Agree	3	Disagree	4	Strongly Disagree	7	Don't Know	8	Refuse to AnswerK78c.	It's OK if we don't follow the method all the time.  (Choose one)	1	Strongly Agree	2	Agree	3	Disagree	4	Strongly Disagree	7	Don't Know	8	Refuse to AnswerIf K70A is equal to 1 or K70C is equal to 1 then skip to K78dIf not, then skip to instruction before K78e.K78d.	It is difficult to refuse sex or use a condom with my partner when I am most fertile.  (Choose one)	1	Strongly Agree	2	Agree	3	Disagree	4	Strongly Disagree	7	Don't Know	8	Refuse to AnswerIf K70B is equal to 1 then skip to K78eIf not, then skip to K100.K78e.	It is difficult for my partner to withdraw before ejaculating every time we have sex.  (Choose one)	1	Strongly Agree	2	Agree	3	Disagree	4	Strongly Disagree	7	Don't Know	8	Refuse to AnswerK100.	Are (Is) you (your partner) using any other methods of contraception?	1	Yes	0	No	7	Don't Know	8	Refuse to AnswerIf (K100 is equal to 0 or K100 is equal to "Don't Know" or K100 is equal to "Refuse to Answer") and (P6 is equal to 2 or P7 is equal to 1), then skip to instruction before K180a.If (K100 is equal to 0 or K100 is equal to "Don't Know" or K100 is equal to "Refuse to Answer") and P6 is equal to 1 and P7 is equal to 0, then skip to K182.K101a.	What is the SECOND method of contraception you are currently using?  (Choose one)	01	Injectables	02	Oral contraceptives (birth control pills)	03	Intrauterine device (IUD; the coil)	04	Implant (put in forearm)	05	Female sterilization (tying woman's tubes)	06	Male sterilization (tying men's tubes)	07	Condoms (for the male)	08	Condoms (for the female)	09	Traditional method [LAM, withdrawal, fertility based awareness (FAMS)/rhythm]	10	Other	98	Refuse to AnswerIf K101a is not equal to 10, then skip to K101b.K101a_s.	Specify other method of secondary contraception:	__ __ __ __ __ __ __ __ __ __ __ __ __ __ __ __ __ __ __ __ __ __ __ __ __ __ __ __ __ __ __ __ __ __ __ __ __ __ __ __ __ __ __ __ __ __ __ __ __ __ __ __ __ __ __ __ __ __ __ __ __ __ __ __ __ __ __ __ __ __ __ __ __ __ __ __ __ __ __ __ __ __ __ __ __ __ __ __ __ __ __ __ __ __ __ __ __ __ __ __K101b.	How long ago did you (your partner) start using this method of contraception?	__ __ __	months	998	Refuse to AnswerIf P7 is equal to 0 and K101a is not equal to 7 and K101a is not equal to 6, then skip to K182.If K101a is equal to 1, then skip to K110.If K101a is equal to 2, then skip to K120.If K101a is equal to 3, then skip to K130a.If K101a is equal to 4, then skip to K140a.If K101a is equal to 5 or K101a is equal to 6, then skip to K150.If K101a is equal to 7 or K101a is equal to 8, then skip to K160.If K101a is equal to 9, then skip to K170.If K101a is equal to 10, then skip to instruction before K180a.Injectable contraceptives-secondaryK110.	With what frequency are the injections recommended?  (Choose one)	1	Every month	2	Every 2 months	3	Every 3 months	4	Every 6 months	8	Refuse to AnswerK111.	How would you describe the timeliness of your last injection?  (Choose one)	1	I received the injection on time	2	I received the injection less than 1 week late	3	I received the injection between 1 and 2 weeks late	4	I received the injection between 2-4 weeks late	5	I received the injection greater than 4 weeks late	6	I did not receive the injection	8	Refuse to AnswerK112a.	In the past 6 months, have you returned to the clinic to receive your contraceptive injection and found that it was unavailable?	1	Yes	0	No	8	Refuse to AnswerIf K112a is equal to 0 or K112a is equal to "Refuse to Answer", then skip to K113.K112b.	What advice did you receive from your provider?  (Choose one)	1	I was referred to another facility to receive my injection	2	I was advised to use condoms	3	I was advised to switch to another method of contraception	4	I was given a date to return to the hospital	5	Other	8	Refuse to AnswerIf K112b is not equal to 5, then skip to K113.K112b_s.	Explain other advice:	__ __ __ __ __ __ __ __ __ __ __ __ __ __ __ __ __ __ __ __ __ __ __ __ __ __ __ __ __ __ __ __ __ __ __ __ __ __ __ __ __ __ __ __ __ __ __ __ __ __ __ __ __ __ __ __ __ __ __ __ __ __ __ __ __ __ __ __ __ __ __ __ __ __ __ __ __ __ __ __ __ __ __ __ __ __ __ __ __ __ __ __ __ __ __ __ __ __ __ __K113.	Have you experienced any side effects from the use of this contraceptive in the past month? If so, how severe were the side effects?  (Choose one)	0	No side effects	1	Minor side effects	2	Moderate side effects	3	Severe side effects	8	Refuse to AnswerPlease indicate the extent to which you agree with each of the following statements.K114a.	I don't want to get pregnant again right now.  (Choose one)	1	Strongly Agree	2	Agree	3	Disagree	4	Strongly Disagree	8	Refuse to AnswerK114b.	It is OK if I miss an injection.  (Choose one)	1	Strongly Agree	2	Agree	3	Disagree	4	Strongly Disagree	8	Refuse to AnswerK114c.	I worry that the injections affect my ability to breastfeed my infant.  (Choose one)	1	Strongly Agree	2	Agree	3	Disagree	4	Strongly Disagree	8	Refuse to AnswerK114d.	I find it difficult to return to the clinic for my injection.  (Choose one)	1	Strongly Agree	2	Agree	3	Disagree	4	Strongly Disagree	8	Refuse to AnswerK114e.	I am confident I will have enough money to continue my contraception.  (Choose one)	1	Strongly Agree	2	Agree	3	Disagree	4	Strongly Disagree	8	Refuse to AnswerK114f.	I worry my contraception may interact with other medications that I am on.  (Choose one)	1	Strongly Agree	2	Agree	3	Disagree	4	Strongly Disagree	8	Refuse to AnswerK114g.	I worry people will find out that I am using contraception injections.  (Choose one)	1	Strongly Agree	2	Agree	3	Disagree	4	Strongly Disagree	8	Refuse to AnswerK114h.	Due to my religious beliefs, I sometimes feel guilty that I am using injectable contraception.  (Choose one)	1	Strongly Agree	2	Agree	3	Disagree	4	Strongly Disagree	8	Refuse to AnswerSkip to instruction before K180a.Oral Contraceptives-secondaryK120.	In the past month, approximately how many days have you missed taking your oral contraceptive?	__ __	days	98	Refuse to AnswerIf K120 is equal to 0 or K120 is equal to "Refuse to Answer", then skip to K122.K121.	Please select the reason(s) why you missed taking the contraceptive pill (select all that apply):  (Check all that apply)	__	I forgot	__	I did not want to take it in front of other people	__	I did not have my oral contraceptives with me	__	I was not feeling well	__	Side effects were too severe	__	Other	__	Refuse to AnswerIf K121F is not equal to 1, then skip to K122.K121f_s.	Specify other reason(s) why missed taking contraceptive pill	__ __ __ __ __ __ __ __ __ __ __ __ __ __ __ __ __ __ __ __ __ __ __ __ __ __ __ __ __ __ __ __ __ __ __ __ __ __ __ __ __ __ __ __ __ __ __ __ __ __ __ __ __ __ __ __ __ __ __ __ __ __ __ __ __ __ __ __ __ __ __ __ __ __ __ __ __ __ __ __ __ __ __ __ __ __ __ __ __ __ __ __ __ __ __ __ __ __ __ __K122.	In the past 6 months, have you run out of your oral contraceptive before you've been able to return to the clinic to refill your prescription.	1	Yes	0	No	8	Refuse to AnswerK123a.	In the past 6 months, have you returned to the clinic to pick up your oral contraceptive prescription and found that it was unavailable?	1	Yes	0	No	8	Refuse to AnswerIf K123a is equal to 0 or K123a is equal to "Refuse to Answer", then skip to K124.K123b.	How many times has this occurred?	__ __	98	Refuse to AnswerK123c.	What advice did you receive from your provider?  (Choose one)	1	I was referred to another facility to fill my prescription	2	I was advised to use condoms	3	I was advised to switch to another method of contraception	4	I was given a date to return to the hospital	5	Other	8	Refuse to AnswerIf K123c is not equal to 5, then skip to K124.K123c_s.	Specify other advice from provider:	__ __ __ __ __ __ __ __ __ __ __ __ __ __ __ __ __ __ __ __ __ __ __ __ __ __ __ __ __ __ __ __ __ __ __ __ __ __ __ __ __ __ __ __ __ __ __ __ __ __ __ __ __ __ __ __ __ __ __ __ __ __ __ __ __ __ __ __ __ __ __ __ __ __ __ __ __ __ __ __ __ __ __ __ __ __ __ __ __ __ __ __ __ __ __ __ __ __ __ __K124.	Have you experienced any side effects from the use of this contraceptive in the past month? If so, how severe were the side effects?  (Choose one)	0	No side effects	1	Minor side effects	2	Moderate side effects	3	Severe side effects	8	Refuse to AnswerPlease indicate what is the best action to be taken in each of the following scenarios.K125a.	You miss one or two days of your pills or start the pack one or two days late:  (Choose one)	1	Discard the missed pill(s) and then continue taking normally the next day	2	Take the missed pill(s) as soon as possible then continue taking normally	3	Throw away the pack and start a new pack after 5 days	4	Take the remaining pills in the pack and start a new pack the next day, with no break	8	Refuse to AnswerK125b.	You miss three or more days in the third week of pills:  (Choose one)	1	Discard the missed pill(s) and then continue taking normally the next day	2	Take the missed pill(s) as soon as possible then continue taking normally	3	Throw away the pack and start a new pack after 5 days	4	Take the remaining pills in the pack and start a new pack the next day, with no break	8	Refuse to AnswerPlease indicate the extent to which you agree with each of the following statements.K126a.	I don't want to get pregnant again right now.  (Choose one)	1	Strongly Agree	2	Agree	3	Disagree	4	Strongly Disagree	8	Refuse to AnswerK126b.	It is difficult to remember to take my pill every day.  (Choose one)	1	Strongly Agree	2	Agree	3	Disagree	4	Strongly Disagree	8	Refuse to AnswerK126c.	It is OK if I forget to take my pills for three days in a row  (Choose one)	1	Strongly Agree	2	Agree	3	Disagree	4	Strongly Disagree	8	Refuse to AnswerK126d.	I worry that my oral contraceptives affect my ability to breastfeed my infant.  (Choose one)	1	Strongly Agree	2	Agree	3	Disagree	4	Strongly Disagree	8	Refuse to AnswerK126e.	I find it difficult to return to the clinic to refill my prescription in time.  (Choose one)	1	Strongly Agree	2	Agree	3	Disagree	4	Strongly Disagree	8	Refuse to AnswerK126f.	I am confident I will have enough money to continue my contraception.  (Choose one)	1	Strongly Agree	2	Agree	3	Disagree	4	Strongly Disagree	8	Refuse to AnswerK126g.	I worry my contraception may interact with other medications that I am on.  (Choose one)	1	Strongly Agree	2	Agree	3	Disagree	4	Strongly Disagree	8	Refuse to AnswerK126h.	I worry people will find out that I am using contraception pills.  (Choose one)	1	Strongly Agree	2	Agree	3	Disagree	4	Strongly Disagree	8	Refuse to AnswerK126i.	Due to my religious beliefs, I sometimes feel guilty that I am using contraception pills.  (Choose one)	1	Strongly Agree	2	Agree	3	Disagree	4	Strongly Disagree	8	Refuse to AnswerSkip to instruction before K180a.Intrauterine Device (IUD)-secondaryK130a.	Approximately how long ago was your IUD inserted:	__ __ __	YEARS	__ __ __	MONTHS	998	Refuse to Answer (Months)K130b.	In approximately how many years should your IUD be removed and replaced?	__ __	years	98	Refuse to AnswerK131.	Have you experienced any side effects from the use of this contraceptive in the past month? If so, how severe were the side effects?  (Choose one)	0	No side effects	1	Minor side effects	2	Moderate side effects	3	Severe side effects	8	Refuse to AnswerIndicate whether each of the following is true or false.K132a.	Women who have not had children cannot use IUD.  (Choose one)	0	False	1	True	7	Don't Know	8	Refuse to AnswerK132b.	An IUD can protect a woman from pregnancy for 12-13 years.  (Choose one)	0	False	1	True	7	Don't Know	8	Refuse to AnswerK132c.	An IUD can migrate to other parts of the body and cause infection.  (Choose one)	0	False	1	True	7	Don't Know	8	Refuse to AnswerK132d.	Women who are breastfeeding can use IUD.  (Choose one)	0	False	1	True	7	Don't Know	8	Refuse to AnswerK132e.	Fertility returns immediately after removal of IUD.  (Choose one)	0	False	1	True	7	Don't Know	8	Refuse to AnswerK132f.	An IUD causes pain and discomfort during intercourse.  (Choose one)	0	False	1	True	7	Don't Know	8	Refuse to AnswerPlease indicate the extent to which you agree with each of the following statements.K133a.	I don't want to get pregnant again right now.  (Choose one)	1	Strongly Agree	2	Agree	3	Disagree	4	Strongly Disagree	8	Refuse to AnswerK133b.	I am confident I will have enough money to continue my contraception.  (Choose one)	1	Strongly Agree	2	Agree	3	Disagree	4	Strongly Disagree	8	Refuse to AnswerK133c.	It will be difficult to continue to use this contraceptive over time.  (Choose one)	1	Strongly Agree	2	Agree	3	Disagree	4	Strongly Disagree	8	Refuse to AnswerK133d.	I worry that my contraception may interact with other medications I am on.  (Choose one)	1	Strongly Agree	2	Agree	3	Disagree	4	Strongly Disagree	8	Refuse to AnswerK133e.	I worry people will find out I am using an IUD.  (Choose one)	1	Strongly Agree	2	Agree	3	Disagree	4	Strongly Disagree	8	Refuse to AnswerK133f.	Due to my religious beliefs, I sometimes feel guilty that I am using an IUD.  (Choose one)	1	Strongly Agree	2	Agree	3	Disagree	4	Strongly Disagree	8	Refuse to AnswerSkip to instruction before K180a.Implants-secondaryK140a.	Approximately how long ago was your implant inserted:	__ __ __	YEARS	__ __ __	MONTHS	998	Refuse to Answer (Months)K140b.	In approximately how many years or months should your implant be removed and replaced?	__ __	YEARS	__ __	MONTHS	98	Refuse to Answer (Years)K141.	Have you experienced any side effects from the use of this contraceptive in the past month? If so, how severe were the side effects?  (Choose one)	0	No side effects	1	Minor side effects	2	Moderate side effects	3	Severe side effects	8	Refuse to AnswerIndicate whether each of the following is true or false.K142a.	Women who have not had children cannot use implants.  (Choose one)	0	False	1	True	7	Don't Know	8	Refuse to AnswerK142b.	An implant can protect a woman from pregnancy for 3-5 years.  (Choose one)	0	False	1	True	7	Don't Know	8	Refuse to AnswerK142c.	An implant can migrate to other parts of the body and cause infection.  (Choose one)	0	False	1	True	7	Don't Know	8	Refuse to AnswerK142d.	Women who are breastfeeding can use implants.  (Choose one)	0	False	1	True	7	Don't Know	8	Refuse to AnswerK142e.	Fertility returns after removal of the implant.  (Choose one)	0	False	1	True	7	Don't Know	8	Refuse to AnswerK142f.	Implants cause pain and discomfort during intercourse.  (Choose one)	0	False	1	True	7	Don't Know	8	Refuse to AnswerPlease indicate the extent to which you agree with each of the following statements.K143a.	I don't want to get pregnant again right now.  (Choose one)	1	Strongly Agree	2	Agree	3	Disagree	4	Strongly Disagree	8	Refuse to AnswerK143b.	I worry my contraceptive implant affects my ability to breastfeed my infant.  (Choose one)	1	Strongly Agree	2	Agree	3	Disagree	4	Strongly Disagree	8	Refuse to AnswerK143c.	I am confident I will have enough money to continue my contraception.  (Choose one)	1	Strongly Agree	2	Agree	3	Disagree	4	Strongly Disagree	8	Refuse to AnswerK143d.	It will be difficult to continue to use this contraceptive over time.  (Choose one)	1	Strongly Agree	2	Agree	3	Disagree	4	Strongly Disagree	8	Refuse to AnswerK143e.	I worry that my contraception may interact with other medications I am on.  (Choose one)	1	Strongly Agree	2	Agree	3	Disagree	4	Strongly Disagree	8	Refuse to AnswerK143f.	I worry people will find out I am using a contraception implant.  (Choose one)	1	Strongly Agree	2	Agree	3	Disagree	4	Strongly Disagree	8	Refuse to AnswerK143g.	Due to my religious beliefs, I sometimes feel guilty that I am using a contraception implant.  (Choose one)	1	Strongly Agree	2	Agree	3	Disagree	4	Strongly Disagree	8	Refuse to AnswerSkip to instruction before K180a.Sterilization-secondaryK150.	Have you experienced any side effects from the use of this contraceptive in the past month? If so, how severe were the side effects?  (Choose one)	0	No side effects	1	Minor side effects	2	Moderate side effects	3	Severe side effects	8	Refuse to AnswerIndicate whether each of the following is true or false.K151a.	Sterilization can be reversed when the couple decides to have another child.  (Choose one)	0	False	1	True	7	Don't Know	8	Refuse to AnswerK151b.	Sterilization can only be performed on women or men with 4 or more living children.  (Choose one)	0	False	1	True	7	Don't Know	8	Refuse to AnswerPlease indicate the extent to which you agree with each of the following statements.K152a.	I don't want (my partner) to get pregnant again right now.  (Choose one)	1	Strongly Agree	2	Agree	3	Disagree	4	Strongly Disagree	8	Refuse to AnswerK152b.	I worry that people will find out about the sterilization procedure.  (Choose one)	1	Strongly Agree	2	Agree	3	Disagree	4	Strongly Disagree	8	Refuse to AnswerIf K101a is equal to 6, then skip to K152d.K152c.	I worry that the sterilization affects my ability to breastfeed my infant.  (Choose one)	1	Strongly Agree	2	Agree	3	Disagree	4	Strongly Disagree	8	Refuse to AnswerK152d.	It is difficult to return to the clinic for follow up care from the sterilization.  (Choose one)	1	Strongly Agree	2	Agree	3	Disagree	4	Strongly Disagree	8	Refuse to AnswerK152e.	Due to my religious beliefs, I sometimes feel guilty about the sterilization.  (Choose one)	1	Strongly Agree	2	Agree	3	Disagree	4	Strongly Disagree	8	Refuse to AnswerSkip to instruction before K180a.Condoms-secondarySunmola, Adegbenga M. "Developing a scale for measuring the barriers to condom use in Nigeria." Bulletin of the World Health Organization 79.10 (2001): 926-932.If P6 is equal to 2 or P7 is equal to 1, then skip to instruction before K180a.If P6 is equal to 1 and P7 is equal to 0, then skip to K182.Traditional Methods-secondaryK170.	Which of the traditional methods were used? (check all that apply)  (Check all that apply)	__	LAM	__	Withdrawal	__	Fertility awareness menstruations strategies (FAMS/rhythm)If K170A is equal to 1, then skip to instruction before K171a.If K170A is equal to 0 and K170C is equal to 1, then skip to K173.If K170A is equal to 0 and K170C is equal to 0 and K170B is equal to 1, then skip to K176.Indicate whether each of the following is true or false.K171a.	My menstrual cycle has not resumed since the birth of my last baby.  (Choose one)	0	False	1	True	7	Don't Know	8	Refuse to AnswerK171b.	I have started feeding my infant other foods and drinks, other than breastmilk.  (Choose one)	0	False	1	True	7	Don't Know	8	Refuse to AnswerK171c.	My baby is not yet 6 months.  (Choose one)	0	False	1	True	7	Don't Know	8	Refuse to AnswerPlease indicate whether LAM is an effective method of contraception in each of the following situations.K172a.	Your infant is less than 6 months of age and on replacement feeding.  (Choose one)	0	Not Effective	1	Effective	7	Don't Know	8	Refuse to AnswerK172b.	Your menses have returned and your infant is exclusively breastfeeding.  (Choose one)	0	Not Effective	1	Effective	7	Don't Know	8	Refuse to AnswerK172c.	You have not started menstruating and your infant is less than 6 months and exclusively breastfeeding.  (Choose one)	0	Not Effective	1	Effective	7	Don't Know	8	Refuse to AnswerK172d.	Your infant is 9 months of age, breastfeeding and starting on solid foods, and your menses have not yet returned.  (Choose one)	0	Not Effective	1	Effective	7	Don't Know	8	Refuse to AnswerIf K170C is equal to 1, then skip to K173.If K170B is equal to 0 and K170C is equal to 0, then skip to instruction before K178a.If K170C is equal to 0 and K170B is equal to 1, then skip to K176.K173.	Please indicate which of the following best describes your menstrual cycle.  (Choose one)	1	My cycle (time between bleeding) is usually less than 26 days	2	My cycle (time between bleeding) is usually between 26-32 days	3	My cycle (time between bleeding) is usually more than 32 days	4	I don't know how long my menstrual cycle usually is	8	Refuse to AnswerK174.	When in her menstrual cycle is a woman most fertile?  (Choose one)	1	When she is menstruating	2	1-7 days after bleeding begins	3	8-19 days after bleeding begins	4	15-21 days after bleeding begins	7	Don't Know	8	Refuse to AnswerK175.	How many fertile days per menstrual cycle must couples avoid unprotected intercourse or abstain from sex to prevent pregnancy while using FAMS?  (Choose one)	1	<5 days	2	8 days	3	10 days	4	12 days	5	14 days	7	Don't Know	8	Refuse to AnswerIf K170B is equal to 0, then skip to instruction before K178a.K176.	In the past 6 months, when having intercourse with your partner, how often have you (your partner) withdrawn before ejaculation?  (Choose one)	0	Never	1	Rarely	2	About half the time	3	Most of the time	4	Always	8	Refuse to AnswerIndicate whether each of the following is true or false.K177.	It is possible for a woman to get pregnant, even if her partner withdraws before ejaculation.  (Choose one)	0	False	1	True	7	Don't Know	8	Refuse to AnswerPlease indicate the extent to which you agree with each of the following statements.K178a.	I don't want to get pregnant again right now  (Choose one)	1	Strongly Agree	2	Agree	3	Disagree	4	Strongly Disagree	7	Don't Know	8	Refuse to AnswerK178b.	I find use of traditional methods disruptive to sex.  (Choose one)	1	Strongly Agree	2	Agree	3	Disagree	4	Strongly Disagree	7	Don't Know	8	Refuse to AnswerK178c.	It's OK if we don't follow the method all the time.  (Choose one)	1	Strongly Agree	2	Agree	3	Disagree	4	Strongly Disagree	7	Don't Know	8	Refuse to AnswerIf K170A is equal to 1 or K170C is equal to 1 then skip to K178dIf not, then skip to instruction before K178e.K178d.	It is difficult to refuse sex or use a condom with my partner when I am most fertile.  (Choose one)	1	Strongly Agree	2	Agree	3	Disagree	4	Strongly Disagree	7	Don't Know	8	Refuse to AnswerIf K170B is equal to 1 then skip to K178eIf not, then skip to instruction before K180a.K178e.	It is difficult for my partner to withdraw before ejaculating every time we have sex.  (Choose one)	1	Strongly Agree	2	Agree	3	Disagree	4	Strongly Disagree	7	Don't Know	8	Refuse to AnswerIndicate on a scale of 1-10, where 1 is not important and 10 is extremely important, how important each of the following factors were when you were selecting your primary method of contraception:K180a.	Changes in weight due to method.	01	Not important	02	03	04	05	Somewhat important	06	07	08	09	10	Extremely important	98	Refuse to AnswerK180b.	Changes in menstruation due to method.	01	Not important	02	03	04	05	Somewhat important	06	07	08	09	10	Extremely important	98	Refuse to AnswerK180c.	Bleeding between periods.	01	Not important	02	03	04	05	Somewhat important	06	07	08	09	10	Extremely important	98	Refuse to AnswerK180d.	Other side effects.	01	Not important	02	03	04	05	Somewhat important	06	07	08	09	10	Extremely important	98	Refuse to AnswerK180e.	Ability to use discretely, so no one knows I use it.	01	Not important	02	03	04	05	Somewhat important	06	07	08	09	10	Extremely important	98	Refuse to AnswerK180f.	Reversibility of method (can be discontinued).	01	Not important	02	03	04	05	Somewhat important	06	07	08	09	10	Extremely important	98	Refuse to AnswerK180g.	Frequency of return visits to the hospital/clinic.	01	Not important	02	03	04	05	Somewhat important	06	07	08	09	10	Extremely important	98	Refuse to AnswerK180h.	How long the method lasts for.	01	Not important	02	03	04	05	Somewhat important	06	07	08	09	10	Extremely important	98	Refuse to AnswerK180i.	Convenience of method.	01	Not important	02	03	04	05	Somewhat important	06	07	08	09	10	Extremely important	98	Refuse to AnswerK180j.	Whether it interrupts sexual intercourse.	01	Not important	02	03	04	05	Somewhat important	06	07	08	09	10	Extremely important	98	Refuse to AnswerK180k.	Protection against HIV or other STDs.	01	Not important	02	03	04	05	Somewhat important	06	07	08	09	10	Extremely important	98	Refuse to AnswerK180l.	Interaction with other medications.	01	Not important	02	03	04	05	Somewhat important	06	07	08	09	10	Extremely important	98	Refuse to AnswerK180m.	Cost of the method.	01	Not important	02	03	04	05	Somewhat important	06	07	08	09	10	Extremely important	98	Refuse to AnswerK180n.	Provider recommendation.	01	Not important	02	03	04	05	Somewhat important	06	07	08	09	10	Extremely important	98	Refuse to AnswerK180o.	Religious acceptability.	01	Not important	02	03	04	05	Somewhat important	06	07	08	09	10	Extremely important	98	Refuse to AnswerK180p.	Partners preference.	01	Not important	02	03	04	05	Somewhat important	06	07	08	09	10	Extremely important	98	Refuse to AnswerPlease indicate the extent to which you agree with each of the following statements.K181a.	My/our provider made the final decision on which contraceptive I/we should use.  (Choose one)	1	Strongly Agree	2	Agree	3	Disagree	4	Strongly Disagree	8	Refuse to AnswerK181b.	My/our provider gave all the support and guidance that I/we needed to successfully use the contraceptive I/we chose.  (Choose one)	1	Strongly Agree	2	Agree	3	Disagree	4	Strongly Disagree	8	Refuse to AnswerK181c.	I am very satisfied with the contraception methods we are using.  (Choose one)	1	Strongly Agree	2	Agree	3	Disagree	4	Strongly Disagree	8	Refuse to AnswerK181d.	I find the contraceptives we are using disruptive to my daily schedule.  (Choose one)	1	Strongly Agree	2	Agree	3	Disagree	4	Strongly Disagree	8	Refuse to AnswerK181e.	I plan to speak to my provider about alternative methods of contraception.  (Choose one)	1	Strongly Agree	2	Agree	3	Disagree	4	Strongly Disagree	8	Refuse to AnswerK181f.	The contraceptive I/we use interferes with the pleasure we experience during sex.  (Choose one)	1	Strongly Agree	2	Agree	3	Disagree	4	Strongly Disagree	8	Refuse to AnswerK182.	Is there another method that you would prefer to use instead of the method(s) you are currently using?	1	Yes	0	No	8	Refuse to AnswerIf (K182 is equal to 0 or K182 is equal to "Refuse to Answer") and P3 is equal to 1, then skip to K185.If (K182 is equal to 0 or K182 is equal to "Refuse to Answer") and P3 is not equal to 1, then skip to K188.K183.	Which other method would you (your partner) prefer to use? (select one)  (Choose one)	01	Injectables	02	Oral contraceptives (birth control pills)	03	Intrauterine device (IUD; the coil)	04	Implant (put in forearm)	05	Female sterilization (tying woman's tubes)	06	Male sterilization (tying men's tubes)	07	Condoms (for the male)	08	Condoms (for the female)	09	Diaphragm	10	Foam/jelly	11	Traditional method (LAM, withdrawal, fertility based awareness/rhythm)	12	Other	98	Refuse to AnswerIf K183 is not equal to 12, then skip to K184.K183a_s.	Specify other preferred method of contraception:	__ __ __ __ __ __ __ __ __ __ __ __ __ __ __ __ __ __ __ __ __ __ __ __ __ __ __ __ __ __ __ __ __ __ __ __ __ __ __ __ __ __ __ __ __ __ __ __ __ __ __ __ __ __ __ __ __ __ __ __ __ __ __ __ __ __ __ __ __ __ __ __ __ __ __ __ __ __ __ __ __ __ __ __ __ __ __ __ __ __ __ __ __ __ __ __ __ __ __ __K184.	For what reason(s) are you not using this method? (check all that apply)  (Check all that apply)	__	Infrequent sex / husband away	__	Infertility/trouble conceiving	__	Not menstruated since last birth	__	Menopausal / hysterectomy	__	Breastfeeding	__	Too many follow-up visits required	__	Partner opposed	__	Others opposed	__	Religious prohibition	__	Side effects	__	Health concerns	__	Lack of access / too far to go to get	__	Costs too much	__	Preferred method not available	__	Inconvenient/ disrupted daily routine	__	Interferes with body's processes	__	Trying to conceive/became pregnant	__	Method failed/conceived while on it	__	Other	__	Don't Know	__	Refuse to AnswerIf K184S is not equal to 1, then skip to instruction before K185.K184_s.	Specify other reason not using this method of contraception:	__ __ __ __ __ __ __ __ __ __ __ __ __ __ __ __ __ __ __ __ __ __ __ __ __ __ __ __ __ __ __ __ __ __ __ __ __ __ __ __ __ __ __ __ __ __ __ __ __ __ __ __ __ __ __ __ __ __ __ __ __ __ __ __ __ __ __ __ __ __ __ __ __ __ __ __ __ __ __ __ __ __ __ __ __ __ __ __ __ __ __ __ __ __ __ __ __ __ __ __If P3 is not equal to 1, then skip to K188.K185.	Have (has) you (your partner) ever used other methods of contraception?	1	Yes	0	No	7	Don't Know	8	Refuse to AnswerIf K185 is equal to 0 or K185 is equal to "Don't Know" or K185 is equal to "Refuse to Answer", then skip to instruction before K197a.K186.	What was the method(s)? (check all that apply)  (Check all that apply)	__	Injectables	__	Oral contraceptives (birth control pills)	__	Intrauterine device (IUD; the coil)	__	Implant (put in forearm)	__	Female sterilization (tying woman's tubes)	__	Male sterilization (tying men's tubes)	__	Condoms (for the male)	__	Condoms (for the female)	__	Diaphragm	__	Foam/jelly	__	Traditional method (LAM, withdrawal, fertility based awareness/rhythm)	__	Other	__	Refuse to AnswerIf K186L is not equal to 1, then skip to K187.K186_s.	Specify other method(s) ever used:	__ __ __ __ __ __ __ __ __ __ __ __ __ __ __ __ __ __ __ __ __ __ __ __ __ __ __ __ __ __ __ __ __ __ __ __ __ __ __ __ __ __ __ __ __ __ __ __ __ __ __ __ __ __ __ __ __ __ __ __ __ __ __ __ __ __ __ __ __ __ __ __ __ __ __ __ __ __ __ __ __ __ __ __ __ __ __ __ __ __ __ __ __ __ __ __ __ __ __ __K187.	What were the reasons for discontinuing this method(s)? (check all that apply)  (Check all that apply)	__	Infrequent sex / husband away	__	Infertility/trouble conceiving	__	Not menstruated since last birth	__	Menopausal / hysterectomy	__	Breastfeeding	__	Too many follow-up visits required	__	Partner opposed	__	Others opposed	__	Religious prohibition	__	Side effects	__	Health concerns	__	Lack of access / too far to go to get	__	Costs too much	__	Preferred method not available	__	Inconvenient/ disrupted daily routine	__	Interferes with body's processes	__	Trying to conceive/became pregnant	__	Method failed/conceived while on it	__	Other	__	Don't Know	__	Refuse to AnswerIf K187S is not equal to 1, then skip to instruction before K188.K187_s.	Specify other reason discontinuing this method of contraception:	__ __ __ __ __ __ __ __ __ __ __ __ __ __ __ __ __ __ __ __ __ __ __ __ __ __ __ __ __ __ __ __ __ __ __ __ __ __ __ __ __ __ __ __ __ __ __ __ __ __ __ __ __ __ __ __ __ __ __ __ __ __ __ __ __ __ __ __ __ __ __ __ __ __ __ __ __ __ __ __ __ __ __ __ __ __ __ __ __ __ __ __ __ __ __ __ __ __ __ __Skip to instruction before K197a.K188.	Did you (your partner) use other contraceptives in the past 6 months, since our last interview, that you (your partner) are no longer using?	1	Yes	0	No	7	Don't Know	8	Refuse to AnswerIf K188 is equal to 0 or K188 is equal to "Don't Know" or K188 is equal to "Refuse to Answer", then skip to instruction before K197a.K189.	What method(s) were these? (check all that apply)  (Check all that apply)	__	Injectables	__	Oral contraceptives (birth control pills)	__	Intrauterine device (IUD; the coil)	__	Implant (put in forearm)	__	Female sterilization (tying woman's tubes)	__	Male sterilization (tying men's tubes)	__	Condoms (for the male)	__	Condoms (for the female)	__	Diaphragm	__	Foam/jelly	__	Traditional method (LAM, withdrawal, fertility based awareness/rhythm)	__	Other	__	Refuse to AnswerIf K189L is not equal to 1, then skip to K190.K189_s.	Specify other method(s) no longer using:	__ __ __ __ __ __ __ __ __ __ __ __ __ __ __ __ __ __ __ __ __ __ __ __ __ __ __ __ __ __ __ __ __ __ __ __ __ __ __ __ __ __ __ __ __ __ __ __ __ __ __ __ __ __ __ __ __ __ __ __ __ __ __ __ __ __ __ __ __ __ __ __ __ __ __ __ __ __ __ __ __ __ __ __ __ __ __ __ __ __ __ __ __ __ __ __ __ __ __ __K190.	What were the reasons for discontinuing this method(s)? (check all that apply)  (Check all that apply)	__	Infrequent sex / husband away	__	Infertility/trouble conceiving	__	Not menstruated since last birth	__	Menopausal / hysterectomy	__	Breastfeeding	__	Too many follow-up visits required	__	Partner opposed	__	Others opposed	__	Religious prohibition	__	Side effects	__	Health concerns	__	Lack of access / too far to go to get	__	Costs too much	__	Preferred method not available	__	Inconvenient/ disrupted daily routine	__	Interferes with body's processes	__	Trying to conceive/became pregnant	__	Method failed/conceived while on it	__	Other	__	Don't Know	__	Refuse to AnswerIf K190S is not equal to 1, then skip to instruction before K191.K190_s.	Specify other reason discontinuing this method of contraception:	__ __ __ __ __ __ __ __ __ __ __ __ __ __ __ __ __ __ __ __ __ __ __ __ __ __ __ __ __ __ __ __ __ __ __ __ __ __ __ __ __ __ __ __ __ __ __ __ __ __ __ __ __ __ __ __ __ __ __ __ __ __ __ __ __ __ __ __ __ __ __ __ __ __ __ __ __ __ __ __ __ __ __ __ __ __ __ __ __ __ __ __ __ __ __ __ __ __ __ __Skip to instruction before K197a.K191.	Why are you and your partner not currently using contraception? (check all that apply)  (Check all that apply)	__	Infrequent sex / husband away	__	Infertility/trouble conceiving	__	Not menstruated since last birth	__	Menopausal / hysterectomy	__	Breastfeeding	__	Too many follow-up visits required	__	Partner opposed	__	Others opposed	__	Religious prohibition	__	Side effects	__	Health concerns	__	Lack of access / too far to go to get	__	Costs too much	__	Preferred method not available	__	Inconvenient/ disrupted daily routine	__	Interferes with body's processes	__	Trying to conceive/became pregnant	__	Method failed/conceived while on it	__	Other	__	Don't Know	__	Refuse to AnswerIf K191S is not equal to 1, then skip to K192.K191_s.	Specify other reason not currently using this method of contraception:	__ __ __ __ __ __ __ __ __ __ __ __ __ __ __ __ __ __ __ __ __ __ __ __ __ __ __ __ __ __ __ __ __ __ __ __ __ __ __ __ __ __ __ __ __ __ __ __ __ __ __ __ __ __ __ __ __ __ __ __ __ __ __ __ __ __ __ __ __ __ __ __ __ __ __ __ __ __ __ __ __ __ __ __ __ __ __ __ __ __ __ __ __ __ __ __ __ __ __ __K192.	Do you plan to initiate contraception at some point?  (Choose one)	0	No	1	Later today	2	Within 1 month	3	Within 6 months	4	Within 1 year	5	In more than 1 year	6	After we have a child	8	Refuse to AnswerK193.	[K193_F]	1	Yes	0	No	7	Don't Know	8	Refuse to AnswerIf K193 is equal to 0 or K193 is equal to "Don't Know" or K193 is equal to "Refuse to Answer", then skip to K196.K194.	What methods were used? (check all that apply)  (Check all that apply)	__	Injectables	__	Oral contraceptives (birth control pills)	__	Intrauterine device (IUD; the coil)	__	Implant (put in forearm)	__	Female sterilization (tying woman's tubes)	__	Male sterilization (tying men's tubes)	__	Condoms (for the male)	__	Condoms (for the female)	__	Diaphragm	__	Foam/jelly	__	Traditional method (LAM, withdrawal, fertility based awareness/rhythm)	__	Other	__	Refuse to AnswerK195.	What were the reasons for discontinuing this method(s)? (check all that apply)  (Check all that apply)	__	Infrequent sex / husband away	__	Infertility/trouble conceiving	__	Not menstruated since last birth	__	Menopausal / hysterectomy	__	Breastfeeding	__	Too many follow-up visits required	__	Partner opposed	__	Others opposed	__	Religious prohibition	__	Side effects	__	Health concerns	__	Lack of access / too far to go to get	__	Costs too much	__	Preferred method not available	__	Inconvenient/ disrupted daily routine	__	Interferes with body's processes	__	Trying to conceive/became pregnant	__	Method failed/conceived while on it	__	Other	__	Refuse to AnswerK196.	[K196_F]	1	Yes	0	No	8	Refuse to AnswerIf K196 is equal to 0 or K196 is equal to "Refuse to Answer", then skip to instruction before L1a.Providers have different styles in dealing with clients and we would like to know more about your experience in receiving support from your provider on the use of contraception. Your responses are confidential and will not be shared with your provider.K197a.	My/our provider clearly explained multiple contraceptive methods to me/us.  (Choose one)	1	Strongly Agree	2	Agree	3	Disagree	4	Strongly Disagree	8	Refuse to AnswerK197b.	The time spent in consultation with my/our provider was sufficient to discuss my/our contraception needs.  (Choose one)	1	Strongly Agree	2	Agree	3	Disagree	4	Strongly Disagree	8	Refuse to AnswerK197c.	My provider gave me/us the opportunity to ask questions and clarify doubts about the contraception I was (we were) considering.  (Choose one)	1	Strongly Agree	2	Agree	3	Disagree	4	Strongly Disagree	8	Refuse to AnswerK197d.	I/we had sufficient privacy when talking with my/our provider about contraception.  (Choose one)	1	Strongly Agree	2	Agree	3	Disagree	4	Strongly Disagree	8	Refuse to AnswerK197e.	My/our provider treats me in a friendly and respectful way when talking about contraception.  (Choose one)	1	Strongly Agree	2	Agree	3	Disagree	4	Strongly Disagree	8	Refuse to AnswerK197f.	My provider gave me choices and options about the contraceptive to use.  (Choose one)	1	Strongly Agree	2	Agree	3	Disagree	4	Strongly Disagree	8	Refuse to AnswerK197g.	My provider understood how I see things with respect to the contraceptive to use.  (Choose one)	1	Strongly Agree	2	Agree	3	Disagree	4	Strongly Disagree	8	Refuse to AnswerK197h.	My provider conveyed confidence in my ability to make changes regarding the contraceptive to use.  (Choose one)	1	Strongly Agree	2	Agree	3	Disagree	4	Strongly Disagree	8	Refuse to AnswerK197i.	My provider listened to how I would like to do things regarding how to prevent pregnancy.  (Choose one)	1	Strongly Agree	2	Agree	3	Disagree	4	Strongly Disagree	8	Refuse to AnswerK197j.	My provider encouraged me to ask questions about contraception.  (Choose one)	1	Strongly Agree	2	Agree	3	Disagree	4	Strongly Disagree	8	Refuse to AnswerK197k.	My provider tried to understand how I see the options to prevent pregnancy.  (Choose one)	1	Strongly Agree	2	Agree	3	Disagree	4	Strongly Disagree	8	Refuse to AnswerKnowledge, attitudes and practices related to SCMThe following questions ask about your knowledge of options to make childbearing safer. Indicate whether you believe each statement is "true", "false", or you "don't know".L1a.	It is possible for an HIV+ woman to have an HIV-negative baby.  (Choose one)	0	False	1	True	7	Don't Know	8	Refuse to AnswerL1b.	HIV antiretrovirals can reduce the risk of passing HIV to a baby.  (Choose one)	0	False	1	True	7	Don't Know	8	Refuse to AnswerL1c.	There are ways to make conception with an HIV-negative partner safer.  (Choose one)	0	False	1	True	7	Don't Know	8	Refuse to AnswerL1d.	Waiting until my virus is fully suppressed will reduce the risk of health complications to the mother during the pregnancy.  (Choose one)	0	False	1	True	7	Don't Know	8	Refuse to AnswerL1e.	Having a sexually transmitted infection will increase the risk of passing HIV to an uninfected partner during unprotected or "live" sex.  (Choose one)	0	False	1	True	7	Don't Know	8	Refuse to AnswerL1f.	There are specific days during a woman's ovulation cycle when she is most fertile (likely to become pregnant).  (Choose one)	0	False	1	True	7	Don't Know	8	Refuse to AnswerL1g.	If an HIV+ person has an "undetectable" amount of HIV virus, it means that person is no longer able to infect someone else.  (Choose one)	0	False	1	True	7	Don't Know	8	Refuse to AnswerL1h.	An HIV-negative male is able to impregnate an HIV-positive woman by ejaculating into a condom or container and then manually injecting the semen into the woman's vagina with a needless syringe.  (Choose one)	0	False	1	True	7	Don't Know	8	Refuse to AnswerL1i.	Only having unprotected sex during the few days each month when the woman is most fertile helps to limit the risk of HIV transmission to an uninfected partner.  (Choose one)	0	False	1	True	7	Don't Know	8	Refuse to AnswerL1j.	There is technology available that can cleanse a man's sperm of the HIV virus.  (Choose one)	0	False	1	True	7	Don't Know	8	Refuse to AnswerL1k.	If an HIV-positive person is consistent with taking their antiretrovirals (ARVs) as prescribed, it is highly unlikely that they can transmit HIV to a sexual partner during intercourse without a condom.  (Choose one)	0	False	1	True	7	Don't Know	8	Refuse to AnswerL1l.	HIV antiretrovirals (ARVs) taken regularly by an HIV-negative partner reduces their risk of getting infected by their HIV+ partner.  (Choose one)	0	False	1	True	7	Don't Know	8	Refuse to AnswerL1m.	If a man is circumcised, he is less likely to be infected by an HIV+ partner during condomless sex.  (Choose one)	0	False	1	True	7	Don't Know	8	Refuse to AnswerL1n.	Using condoms can prevent an unwanted pregnancy.  (Choose one)	0	False	1	True	7	Don't Know	8	Refuse to AnswerL1o.	Using modern contraceptives such as implants, DEPO infections, IUD, and contraceptive pills can prevent unwanted pregnancies.  (Choose one)	0	False	1	True	7	Don't Know	8	Refuse to AnswerL1p.	Female sterilization (tying of tubes) is a form of contraception that can prevent unwanted pregnancies.  (Choose one)	0	False	1	True	7	Don't Know	8	Refuse to AnswerL1q.	Male sterilization (tying of tubes) is a form of contraception that can prevent unwanted pregnancies.  (Choose one)	0	False	1	True	7	Don't Know	8	Refuse to AnswerL1r.	Traditional contraceptive methods such as withdrawing before ejaculation, the rhythm method or cervical mucus method are less reliable than modern contraceptives in preventing unwanted pregnancies.  (Choose one)	0	False	1	True	7	Don't Know	8	Refuse to AnswerIf I9 is equal to 1, then skip to instruction before L2.L1s.	Have you and your partner been trying to conceive a child at any time in the past 6 months?	1	Yes	0	No	8	Refuse to AnswerIf L1s is equal to 0 or L1s is equal to "Refuse to Answer", then skip to L5.INTERVIEWER: For male respondents, if their female partner is available to respond to questions, she should answer L2-L4i (alone, without male participant in the room).If B6 is equal to 0 or B6 is equal to 1 then Remember, like all questions I am asking you today about your partner, the next several questions pertain to the partner you had at the start of the study..L2.	In the past 6 months, have you used any method to reduce risk of HIV transmission while trying to conceive a child with your partner?	1	Yes	0	No	8	Refuse to AnswerL2a.	In the past 6 months, have you only had unprotected or "live" sex on the specific days each month in which you (your partner) are/is most fertile? (TUI)	1	Yes	0	No	8	Refuse to AnswerIf (L2a is equal to 0 or L2a is equal to "Refuse to Answer") and P6 is equal to 1, then skip to L3a.If (L2a is equal to 0 or L2a is equal to "Refuse to Answer") and P6 is equal to 2, then skip to L4a.L2b.	Can you describe in detail how you used this method?  NTERVIEWER: Depending on what the respondent says, use the following probes to elicit more info if the respondent does not mention these things on their own. Provide client with a calendar when asking questions related to timing of period and most fertile days.  How do you determine when the most fertile days are?  What was the first day of your (your partners) last period (started to  - menstruate or bleed)?  How many days is your (partners) ovulation cycle?  How many days in the most fertile period?  On what date(s) did you have unprotected sex?  Do you ever use condoms? If respondent says Yes: When or how often are condoms used?	__ __ __ __ __ __ __ __ __ __ __ __ __ __ __ __ __ __ __ __ __ __ __ __ __ __ __ __ __ __ __ __ __ __ __ __ __ __ __ __ __ __ __ __ __ __ __ __ __ __ __ __ __ __ __ __ __ __ __ __ __ __ __ __ __ __ __ __ __ __ __ __ __ __ __ __ __ __ __ __ __ __ __ __ __ __ __ __ __ __ __ __ __ __ __ __ __ __ __ __ __ __ __ __ __ __ __ __ __ __ __ __ __ __ __ __ __ __ __ __ __ __ __ __ __ __ __ __ __ __ __ __ __ __ __ __ __ __ __ __ __ __ __ __ __ __ __ __ __ __ __ __ __ __ __ __ __ __ __ __ __ __ __ __ __ __ __ __ __ __ __ __ __ __ __ __ __ __ __ __ __ __ __ __ __ __ __ __ __ __ __ __ __ __ __ __ __ __ __ __ __ __ __ __ __ __ __ __ __ __ __ __ __ __ __ __ __ __ __ __ __ __ __ __ __ __ __ __ __ __ __ __ __ __ __ __ __ __ __ __ __ __ __ __ __ __ __ __ __ __INTERVIEWER: Rate the following components of using TUI (0=not present; 1 = present; 2= insufficient information to rate)L2b1.	Reports the method used to determine timing of the most fertile days (e.g., calendar, mucous stretchiness, text from provider, moon beads) (Choose one)	0	Not present	1	Present	2	Insufficient information to rate	8	Refuse to AnswerRecord methods used:L2b1a. Calendar. 0=no, 1=yesL2b1b. Phone calls or SMS texts from nurse. 0=no, 1=yesL2b1c. Mucous stretchiness. 0=no, 1=yesL2b1d. Moon beads. 0=no, 1=yesL2b1e. Other method:  please specify:  _________________________ L2b2.	Knows the date of last period.  (Choose one)	0	Not present	1	Present	2	Insufficient information to rate	8	Refuse to AnswerL2b3.	Knows length of the ovulation cycle.  (Choose one)	0	Not present	1	Present	2	Insufficient information to rate	8	Refuse to AnswerL2b4.	Knows how to accurately determine the timing of the most fertile days (e.g., if 28 day cycle, and ovulation starts at day 14, than most fertile period is from day 13 to 15).  (Choose one)	0	Not present	1	Present	2	Insufficient information to rate	8	Refuse to AnswerL2b5.	Knows that most fertile period is 3 days.  (Choose one)	0	Not present	1	Present	2	Insufficient information to rate	8	Refuse to AnswerL2b6.	Reports having unprotected intercourse during the 3 most fertile days.  (Choose one)	0	Not present	1	Present	2	Insufficient information to rate	8	Refuse to AnswerL2b7.	Reports always using condoms during sex that takes place outside the 3 most fertile days.  (Choose one)	0	Not present	1	Present	2	Insufficient information to rate	8	Refuse to AnswerL2b8.	Interviewer rating of use:  (Choose one)	0	Inaccurate use	1	Fully accurate use	8	Refuse to AnswerL2c.	Please rate the level of difficulty in using this method.  (Choose one)	0	Not at all difficult	1	Somewhat difficult	2	Very difficult	8	Refuse to AnswerL2d.	How many months of the past 6 months did you use this method?	__	months	8	Refuse to AnswerL2e.	Once you started using this method, did you ever stop using the method?	1	Yes	0	No	8	Refuse to AnswerIf (L2e is equal to 0 or L2e is equal to "Refuse to Answer") and (I1 is equal to 0 or I1 is equal to 2 or I1 is equal to "Refuse to Answer" or I1 is equal to "skipped") and P6 is equal to 1, then skip to L3a.If (L2e is equal to 0 or L2e is equal to "Refuse to Answer") and (I1 is equal to 0 or I1 is equal to 2 or I1 is equal to "Refuse to Answer" or I1 is equal to "skipped") and P6 is equal to 2, then skip to L4a.If (L2e is equal to 0 or L2e is equal to "Refuse to Answer") and I1 is equal to 1, then skip to L2i.L2f.	After how many months of use did you stop?	__	months	8	Refuse to AnswerL2g.	What were the reasons for stopping your use of the method? (Check all that apply)  (Check all that apply)	__	Became pregnant	__	I didn't like the method	__	My partner didn't like the method	__	The method was too difficult	__	We chose to use another method	__	Other	__	Refuse to AnswerIf L2gF is not equal to 1, then skip to L2h.L2g_s.	Specify other reasons for stopping your use of the method:	__ __ __ __ __ __ __ __ __ __ __ __ __ __ __ __ __ __ __ __ __ __ __ __ __ __ __ __ __ __ __ __ __ __ __ __ __ __ __ __ __ __ __ __ __ __ __ __ __ __ __ __ __ __ __ __ __ __ __ __ __ __ __ __ __ __ __ __ __ __ __ __ __ __ __ __ __ __ __ __ __ __ __ __ __ __ __ __ __ __ __ __ __ __ __ __ __ __ __ __L2h.	Did you restart the use of this method after stopping?	1	Yes	0	No	8	Refuse to AnswerIf (I1 is equal to 0 or I1 is equal to 2 or I1 is equal to "Refuse to Answer" or I1 is equal to "skipped") and P6 is equal to 1, then skip to L3a.If (I1 is equal to 0 or I1 is equal to 2 or I1 is equal to "Refuse to Answer" or I1 is equal to "skipped") and P6 is equal to 2, then skip to L4a.If I1 is equal to 1, then skip to L2i.L2i.	Did you use this method prior to learning of your recent pregnancy?	1	Yes	0	No	8	Refuse to AnswerIf P6 is equal to 2, then skip to L4a.L3a.	In the past 6 months, have you used technology that cleanses your sperm or semen of the HIV virus? (sperm washing) 	1	Yes	0	No	8	Refuse to AnswerIf (L3a is equal to 0 or L3a is equal to "Refuse to Answer") and (L2a is equal to 0 or L2a is equal to "Refuse to Answer"), then skip to L5.If (L3a is equal to 0 or L3a is equal to "Refuse to Answer") and L2a is equal to 1, then skip to L5.L3b.	Can you describe in detail how you used this method and where you received the service? nterviewer will look for following details to rate use:   Service was received at one of the clinics providing sperm washing  The man abstains from sex for 3-5 days prior to procedure  During fertile period, male ejaculated into condom or other form of containment  The health provider (HP) takes the semen and uses a procedure to cleanse it  HP makes the woman lie on the bed and uses some metal to open the vagina and uses some tube to insert semen into vagina   Insertion of semen into woman takes place during her most fertile days of cycle  Use of condoms on all days outside of that most fertile period	__ __ __ __ __ __ __ __ __ __ __ __ __ __ __ __ __ __ __ __ __ __ __ __ __ __ __ __ __ __ __ __ __ __ __ __ __ __ __ __ __ __ __ __ __ __ __ __ __ __ __ __ __ __ __ __ __ __ __ __ __ __ __ __ __ __ __ __ __ __ __ __ __ __ __ __ __ __ __ __ __ __ __ __ __ __ __ __ __ __ __ __ __ __ __ __ __ __ __ __ __ __ __ __ __ __ __ __ __ __ __ __ __ __ __ __ __ __ __ __ __ __ __ __ __ __ __ __ __ __ __ __ __ __ __ __ __ __ __ __ __ __ __ __ __ __ __ __ __ __ __ __ __ __ __ __ __ __ __ __ __ __ __ __ __ __ __ __ __ __ __ __ __ __ __ __ __ __ __ __ __ __ __ __ __ __ __ __ __ __ __ __ __ __ __ __ __ __ __ __ __ __ __ __ __ __ __ __ __ __ __ __ __ __ __ __ __ __ __ __ __ __ __ __ __ __ __ __ __ __ __ __ __ __ __ __ __ __ __ __ __ __ __ __ __ __ __ __ __ __L3b1.	Interviewer rating of use:  (Choose one)	0	Inaccurate use	1	Fully accurate use	8	Refuse to AnswerL3c.	Please rate the level of difficulty in using this method.  (Choose one)	0	Not at all difficult	1	Somewhat difficult	2	Very difficult	8	Refuse to AnswerL3d.	When was this procedure conducted?	__ __	months	98	Refuse to AnswerL3e.	Where was the procedure conducted?	__ __ __ __ __ __ __ __ __ __ __ __ __ __ __ __ __ __ __ __ __ __ __ __ __ __ __ __ __ __ __ __ __ __ __ __ __ __ __ __ __ __ __ __ __ __ __ __ __ __ __ __ __ __ __ __ __ __ __ __ __ __ __ __ __ __ __ __ __ __ __ __ __ __ __ __ __ __ __ __ __ __ __ __ __ __ __ __ __ __ __ __ __ __ __ __ __ __ __ __Skip to L5.L4a.	In the past 6 months, has your partner ejaculated into a condom or container and then manually injected the semen into your vagina? (MSI)	1	Yes	0	No	8	Refuse to AnswerIf (L4a is equal to 0 or L4a is equal to "Refuse to Answer") and (L2a is equal to 0 or L2a is equal to "Refuse to Answer"), then skip to L5.If (L4a is equal to 0 or L4a is equal to "Refuse to Answer") and L2a is equal to 1, then skip to L5.L4b.	Can you describe in detail how you used this method?  NTERVIEWER: Depending on what the respondent says, use the following probes to elicit more info if the respondent does not mention these things on their own. Provide client with a calendar when asking questions related to timing of ovulation and most fertile days.  How do you determine when the most fertile days are?  What was the first day of your (your partners) last period (started to menstruate or bleed)?  How many days is your (partners) ovulation cycle?  How many days in the most fertile period?  On what date(s) did you inject semen into the vagina?   How long after ejaculation was the semen injected into the vagina?  What was used to inject the semen into the vagina?   When the semen was injected, what position was the woman in?  For how long did the woman stay in this position after the semen was injected?  Do you ever use condoms during sexual intercourse? If respondent says Yes: When or how often are condoms used?	__ __ __ __ __ __ __ __ __ __ __ __ __ __ __ __ __ __ __ __ __ __ __ __ __ __ __ __ __ __ __ __ __ __ __ __ __ __ __ __ __ __ __ __ __ __ __ __ __ __ __ __ __ __ __ __ __ __ __ __ __ __ __ __ __ __ __ __ __ __ __ __ __ __ __ __ __ __ __ __ __ __ __ __ __ __ __ __ __ __ __ __ __ __ __ __ __ __ __ __ __ __ __ __ __ __ __ __ __ __ __ __ __ __ __ __ __ __ __ __ __ __ __ __ __ __ __ __ __ __ __ __ __ __ __ __ __ __ __ __ __ __ __ __ __ __ __ __ __ __ __ __ __ __ __ __ __ __ __ __ __ __ __ __ __ __ __ __ __ __ __ __ __ __ __ __ __ __ __ __ __ __ __ __ __ __ __ __ __ __ __ __ __ __ __ __ __ __ __ __ __ __ __ __ __ __ __ __ __ __ __ __ __ __ __ __ __ __ __ __ __ __ __ __ __ __ __ __ __ __ __ __ __ __ __ __ __ __ __ __ __ __ __ __ __ __ __ __ __ __INTERVIEWER: Rate the following components of using MSI (0=not present; 1 = present; 2= insufficient information to rate)L4b1.	Reports the method used to determine timing of the most fertile days (e.g., calendar, mucous stretchiness, text from provider, moon beads).  (Choose one)	0	Not present	1	Present	2	Insufficient information to rate	8	Refuse to AnswerRecord methods used:L4b1a. Calendar. 0=no, 1=yesL4b1b. Phone calls or SMS texts from nurse. 0=no, 1=yesL4b1c. Mucous stretchiness. 0=no, 1=yesL4b1d. Moon beads. 0=no, 1=yesL4b1e. Other method:  please specify:  _________________________ L4b2.	Knows the date of last period.  (Choose one)	0	Not present	1	Present	2	Insufficient information to rate	8	Refuse to AnswerL4b3.	Knows length of the ovulation cycle.  (Choose one)	0	Not present	1	Present	2	Insufficient information to rate	8	Refuse to AnswerL4b4.	Knows how to accurately determine the timing of the most fertile days (e.g., half way through woman's cycle; if 28 day cycle, and ovulation starts at day 14, than most fertile period is from day 13 to 15).  (Choose one)	0	Not present	1	Present	2	Insufficient information to rate	8	Refuse to AnswerL4b5.	Knows that most fertile period is 3 days.  (Choose one)	0	Not present	1	Present	2	Insufficient information to rate	8	Refuse to AnswerL4b6.	Man collected semen using condom during sex or a cup/container.  (Choose one)	0	Not present	1	Present	2	Insufficient information to rate	8	Refuse to AnswerL4b7.	Syringe was used to inject semen into vagina.  (Choose one)	0	Not present	1	Present	2	Insufficient information to rate	8	Refuse to AnswerL4b8.	Injected semen within one hour after ejaculation.  (Choose one)	0	Not present	1	Present	2	Insufficient information to rate	8	Refuse to AnswerL4b9.	Semen was injected into vagina during the 3 most fertile days.  (Choose one)	0	Not present	1	Present	2	Insufficient information to rate	8	Refuse to AnswerL4b10.	Woman was in proper position (on back with hips raised).  (Choose one)	0	Not present	1	Present	2	Insufficient information to rate	8	Refuse to AnswerL4b11.	Woman remained in proper position for at least 30 minutes after injection of semen.  (Choose one)	0	Not present	1	Present	2	Insufficient information to rate	8	Refuse to AnswerL4b12.	Reports always using condoms during sexual intercourse.  (Choose one)	0	Not present	1	Present	2	Insufficient information to rate	8	Refuse to AnswerL4b13.	Interviewer rating of use:  (Choose one)	0	Inaccurate use	1	Fully accurate use	8	Refuse to AnswerL4c.	Please rate the level of difficulty in using this method.  (Choose one)	0	Not at all difficult	1	Somewhat difficult	2	Very difficult	8	Refuse to AnswerL4d.	How many months of the past 6 months did you use this method?	__	months	8	Refuse to AnswerL4e.	Once you started using this method, did you ever stop using the method?	1	Yes	0	No	8	Refuse to AnswerIf (L4e is equal to 0 or L4e is equal to "Refuse to Answer") and (I1 is equal to 0 or I1 is equal to 2 or I1 is equal to "Refuse to Answer" or I1 is equal to "skipped"), then skip to L5.If (L4e is equal to 0 or L4e is equal to "Refuse to Answer") and I1 is equal to 1, then skip to L4i.L4f.	After how many months of use did you stop?	__	months	8	Refuse to AnswerL4g.	What were the reasons for stopping your use of the method? (Check all that apply)  (Check all that apply)	__	Became pregnant	__	I didn't like the method	__	My partner didn't like the method	__	The method was too difficult	__	We chose to use another method	__	Other	__	Refuse to AnswerIf L4gF is not equal to 1, then skip to L4h.L4g_s.	Specify other reasons for stopping your use of the method:	__ __ __ __ __ __ __ __ __ __ __ __ __ __ __ __ __ __ __ __ __ __ __ __ __ __ __ __ __ __ __ __ __ __ __ __ __ __ __ __ __ __ __ __ __ __ __ __ __ __ __ __ __ __ __ __ __ __ __ __ __ __ __ __ __ __ __ __ __ __ __ __ __ __ __ __ __ __ __ __ __ __ __ __ __ __ __ __ __ __ __ __ __ __ __ __ __ __ __ __L4h.	Did you restart the use of this method after stopping?	1	Yes	0	No	8	Refuse to AnswerIf I1 is equal to 0 or I1 is equal to 2 or I1 is equal to "Refuse to Answer" or I1 is equal to "skipped", then skip to L5.If I1 is equal to 1, then skip to L4i.L4i.	Did you use this method prior to learning of your recent pregnancy?	1	Yes	0	No	8	Refuse to AnswerL5.	[L5_F]	1	Yes	0	No	8	Refuse to AnswerIf (L5 is equal to 0 or L5 is equal to "Refuse to Answer") and (L1s is equal to 0 or L1s is equal to "Refuse to Answer"), then skip to instruction before L15a.If (L5 is equal to 0 or L5 is equal to "Refuse to Answer") and (L2a is equal to 1 or L3a is equal to 1 or L4a is equal to 1), then skip to instruction before L6.Provider assistance for making childbearing decisions daptation of the Health Care Climate Questionnaire (HCCQ); Williams GC, McGregor HA, and Sharp D, et al. Testing a self-determination theory intervention for motivating tobacco cessation: supporting autonomy and competence in a clinical trial. Health Psychol. Jan 2006;25(1):91-101.In the past 6 months, have you used any of these methods to reduce risk of HIV transmission while trying to conceive a child with your partner:L6.	Have you delayed trying to conceive until you had a higher CD4 count?	1	Yes	0	No	8	Refuse to AnswerL7.	Have you delayed trying to conceive until your HIV viral load was undetectable?	1	Yes	0	No	8	Refuse to AnswerL8.	Have you started ART earlier than you would have otherwise?	1	Yes	0	No	8	Refuse to AnswerL9.	Has your partner taken HIV antiretroviral medication (Truvada; PrEP) on most days during the months in which you were trying to conceive?	1	Yes	0	No	8	Refuse to AnswerL10a.	Have you been tested for sexually transmitted infections in the past 6 months?	1	Yes	0	No	8	Refuse to AnswerL10b.	Has your partner been tested for sexually transmitted infections in the past 6 months?	1	Yes	0	No	7	Don't Know	8	Refuse to AnswerL11a1.	Have you been diagnosed with any sexually transmitted infections (other than HIV) in the past 6 months?	1	Yes	0	No	7	Don't Know	8	Refuse to AnswerL11a2.	Has your partner been diagnosed with any sexually transmitted infections (other than HIV) in the past 6 months?	1	Yes	0	No	7	Don't Know	8	Refuse to AnswerIf L11a1 is equal to 0 or L11a1 is equal to "Don't Know" or L11a1 is equal to "Refuse to Answer", then skip to instruction before L11b2.L11b1.	Has your infection(s) been treated?	1	Yes	0	No	7	Don't Know	8	Refuse to AnswerIf (L11a2 is equal to 0 or L11a2 is equal to "Don't Know" or L11a2 is equal to "Refuse to Answer") and P6 is equal to 1, then skip to instruction before L13a.If (L11a2 is equal to 0 or L11a2 is equal to "Don't Know" or L11a2 is equal to "Refuse to Answer") and P6 is equal to 2, then skip to instruction before L12.L11b2.	Has your partners infection(s) been treated?	1	Yes	0	No	7	Don't Know	8	Refuse to AnswerIf P6 is equal to 1, then skip to instruction before L13a.L12.	Has your partner been circumcised?	1	Yes	0	No	8	Refuse to AnswerSCM MotivationScale adapted from Gerkovich M, Williams K, Catley D, Goggin K. Development and Validation of a Scale to Measure Motivation to Adhere to HIV Medication. IAPAC Poster Presentation. Miami, Florida, May 23, 2008. Please use the following scale of 0 to 10 to indicate how confident you are that you could perform each of the tasks described below.SCM Self-efficacyScale adapted from Johnson M, Neilands T, Dilworth S, Morin S, Remien R, ChesneyM. The Role of Self-Efficacy in HIV Treatment Adherence: Validation of the HIV Treatment Adherence Self-Efficacy Scale (HIV-ASES). J Behav Med. 2007 Jun 23.SCM cultural acceptabilityThe following statements are about the cultural acceptability of using safer conception strategies to help people affected by HIV have children safely. Please identify the extent to which you agree or disagree with these statements.L15a.	Couples living with HIV are willing to restrict unprotected sex to only 3 specific days per month, when the woman is most fertile, if it helps to conceive a child more safely.  (Choose one)	1	Strongly Agree	2	Agree	3	Disagree	4	Strongly Disagree	8	Refuse to AnswerL15b.	HIV negative partners of HIV+ patients would be willing to take HIV medications every day during the months in which they were trying to conceive in order to reduce their risk of infection.  (Choose one)	1	Strongly Agree	2	Agree	3	Disagree	4	Strongly Disagree	8	Refuse to AnswerL15c.	HIV negative male partners of HIV+ women would be willing to be circumcised in order to reduce the risk of becoming HIV infected during unprotected sexual intercourse when attempting to conceive.  (Choose one)	1	Strongly Agree	2	Agree	3	Disagree	4	Strongly Disagree	8	Refuse to AnswerL15d.	In couples where the man is HIV negative and the woman is HIV+, the man would be willing to ejaculate into a container or condom, so that this semen could be inserted into the woman's vagina with a syringe in order to remove any risk of him becoming infected while trying to impregnate her.  (Choose one)	1	Strongly Agree	2	Agree	3	Disagree	4	Strongly Disagree	8	Refuse to AnswerL15e.	In couples where the man is HIV negative and the woman is HIV+, the woman would be willing to allow the man to ejaculate into a container or condom, and then have this semen inserted into her vagina with a syringe in order to remove any risk of him becoming infected while trying to impregnate her.  (Choose one)	1	Strongly Agree	2	Agree	3	Disagree	4	Strongly Disagree	8	Refuse to AnswerL15f.	If cost is not an issue, couples in which the man is HIV+ and the woman is not infected would be willing to have the man's sperm cleansed of HIV using technological procedures and then inserted into the woman's vagina using a syringe, in order to reduce the risk of her becoming infected.  (Choose one)	1	Strongly Agree	2	Agree	3	Disagree	4	Strongly Disagree	8	Refuse to AnswerPerceived cultural pressure to have childrenThe following statements are about the cultural importance of childbearing. Please identify the extent to which you agree or disagree with these statements.M1a.	An adult female is not considered a true woman until she has at least one child.  (Choose one)	1	Strongly Disagree	2	Somewhat Disagree	3	Somewhat Agree	4	Strongly Agree	8	Refuse to AnswerM1b.	An adult male is not considered a true man until he has at least one child.  (Choose one)	1	Strongly Disagree	2	Somewhat Disagree	3	Somewhat Agree	4	Strongly Agree	8	Refuse to AnswerM1c.	It is very important that a married couple has children together in order to legitimize the relationship.  (Choose one)	1	Strongly Disagree	2	Somewhat Disagree	3	Somewhat Agree	4	Strongly Agree	8	Refuse to AnswerM1d.	Family members often pressure a couple to have children.  (Choose one)	1	Strongly Disagree	2	Somewhat Disagree	3	Somewhat Agree	4	Strongly Agree	8	Refuse to AnswerM1e.	If family members know that a member of the couple is HIV-positive, the family will not expect them to have a child.  (Choose one)	1	Strongly Disagree	2	Somewhat Disagree	3	Somewhat Agree	4	Strongly Agree	8	Refuse to AnswerPlease indicate the extent to which you agree or disagree with each of the following statements.M2a.	I/we cannot become pregnant while I am (she is) breastfeeding, even if I have (she had) started menstruating again.  (Choose one)	1	Strongly Agree	2	Agree	3	Disagree	4	Strongly Disagree	8	Refuse to AnswerM2b.	Contraceptive use contributes to promiscuity in women.  (Choose one)	1	Strongly Agree	2	Agree	3	Disagree	4	Strongly Disagree	8	Refuse to AnswerM2c.	Contraceptive use decreases a woman's sexual desire.  (Choose one)	1	Strongly Agree	2	Agree	3	Disagree	4	Strongly Disagree	8	Refuse to AnswerM2d.	Hormonal contraception is dangerous for women's health.  (Choose one)	1	Strongly Agree	2	Agree	3	Disagree	4	Strongly Disagree	8	Refuse to AnswerM2e.	Hormonal contraception is safe to use while breastfeeding.  (Choose one)	1	Strongly Agree	2	Agree	3	Disagree	4	Strongly Disagree	8	Refuse to AnswerM2f.	Hormonal contraception can cause permanent sterility in women.  (Choose one)	1	Strongly Agree	2	Agree	3	Disagree	4	Strongly Disagree	8	Refuse to AnswerM2g.	Hormonal contraception can cause birth defects in infants.  (Choose one)	1	Strongly Agree	2	Agree	3	Disagree	4	Strongly Disagree	8	Refuse to AnswerM2h.	My religion supports the use of contraception.  (Choose one)	1	Strongly Agree	2	Agree	3	Disagree	4	Strongly Disagree	8	Refuse to AnswerM2i.	I (my partner) need(s) the permission of my partner (my permission) before I (she) starts contraception.  (Choose one)	1	Strongly Agree	2	Agree	3	Disagree	4	Strongly Disagree	8	Refuse to AnswerM2j.	Contraceptives should only be used by married women.  (Choose one)	1	Strongly Agree	2	Agree	3	Disagree	4	Strongly Disagree	8	Refuse to AnswerM2k.	I worry that long term methods - like intrauterine devices and implants - are painful to use.  (Choose one)	1	Strongly Agree	2	Agree	3	Disagree	4	Strongly Disagree	8	Refuse to AnswerM2l.	My community does not support the use of contraceptives.  (Choose one)	1	Strongly Agree	2	Agree	3	Disagree	4	Strongly Disagree	8	Refuse to AnswerM2m.	The side effects of modern contraceptives outweigh their benefits.  (Choose one)	1	Strongly Agree	2	Agree	3	Disagree	4	Strongly Disagree	8	Refuse to AnswerConsistent condom useN1.	In the last 6 months, how often have you and your partner had intercourse?  (Choose one)	0	Never	1	Rarely (about once a month or less)	2	A few times a month	3	Once a week	4	Several times a week	8	Refuse to AnswerN1a.	Have you and your partner been available to each other for most of the past 6 months, or has one of you been away for work or other reasons for significant periods of time?  (Choose one)	0	Yes, there's been periods of time where we have not been together	1	No, there have not been any lengthy periods where we were not together	8	Refuse to AnswerN1b.	Have you (your partner) had any problems with erectile dysfunction or keeping an erection during sex, in the last 6 months?	1	Yes	0	No	8	Refuse to AnswerIf (N1 is equal to 0 or N1 is equal to "Refuse to Answer") and I1 is equal to 1, then skip to instruction before N5.If (N1 is equal to 0 or N1 is equal to "Refuse to Answer") and (I1 is equal to 0 or I1 is equal to 2 or I1 is equal to "Refuse to Answer" or I1 is equal to "skipped"), then skip to O1.N2.	Thinking of all the times you had intercourse with your partner IN THE LAST 6 MONTHS, how often did you use a condom?  (Choose one)	0	Never	1	Sometimes	2	Always	8	Refuse to AnswerIf N2 is equal to 0 or N2 is equal to "Refuse to Answer", then skip to N4.N3.	Thinking of THE LAST TIME you had intercourse with your partner, did you (your partner) use a condom?	1	Yes	0	No	8	Refuse to AnswerN4.	Has the desire for wanting a child contributed to you and your partner not using condoms during intercourse over the past 6 months?	1	Yes	0	No	8	Refuse to AnswerIf I1 is equal to 0 or I1 is equal to 2 or I1 is equal to "Refuse to Answer" or I1 is equal to "skipped", then skip to O1.Now I want you to think about the period of time since you learned of your (partner's) recent pregnancy.N5.	Since learning of your (partner's) recent pregnancy, how often have you and your partner had intercourse?  (Choose one)	0	Never	1	Rarely (1-2 per month over past 3 months)	2	A few times a month	3	Once a week	4	Several times a week	8	Refuse to AnswerIf N5 is equal to 0 or N5 is equal to "Refuse to Answer", then skip to O1.N6.	Since learning of the pregnancy, how often do you and your partner use a condom when having sexual intercourse with each other?  (Choose one)	0	Never	1	Sometimes	2	Always	8	Refuse to AnswerReceipt of FP/SCC servicesO1.	In the past 6 months, have you discussed issues related to future childbearing with a clinic provider?	1	Yes	0	No	8	Refuse to AnswerIf O1 is equal to 0 or O1 is equal to "Refuse to Answer", then skip to O3.O2.	Did this discussion include any of the following? (Please select all that apply)  (Check all that apply)	__	Transmission risks to sexual partner	__	Transmission risks to the child	__	Health factors that contribute to a healthy pregnancy	__	PMTCT services available	__	Safer conception methods that can be used	__	How HIV antiretrovirals (ARVs) can improve safety of pregnancy	__	How your HIV disease (CD4 and viral load) plays a role in safety of pregnancy	__	Provider judgment regarding your desire to have a child	__	Support regarding the desire to have a child	__	Methods for preventing pregnancy (contraception)	__	Other	__	Refuse to AnswerO3.	Do you think childbearing and your needs related to your decision of whether or not to have a child is a topic your health care providers are willing to discuss?	1	Yes	0	No	8	Refuse to AnswerO4.	Have your providers explained the safer conception methods that are available for you and your partner, if you were to decide that you wanted to have a child?  (Choose one)	0	No, my providers have not talked to me about these methods at all	1	Yes, my providers have explained these methods to me somewhat	2	Yes, my providers have explained these methods to me very well	8	Refuse to AnswerO5.	How would you rate the quality of services at this clinic regarding your childbearing needs, whether those needs are related to having a child safely or preventing pregnancy?  (Choose one)	1	Very poor	2	Poor	3	Fair	4	Good	5	Very good	8	Refuse to AnswerIf P6 is not equal to 1, then skip to instruction before O6c.  THIS SHOULD BE If P3 is not equal to 1O6a.	Have you EVER had counseling sessions with a provider in which you talked about decision making related to whether or not to have a child?	1	Yes	0	No	8	Refuse to AnswerO6b.	Have you EVER had counseling sessions with a provider in which you received instruction and support for how to use methods to reduce risks of transmitting HIV infection to your partner while trying to conceive a child?	1	Yes	0	No	8	Refuse to AnswerIn the past 6 months, how many counseling sessions or clinic visits with a provider have you had in which you talked about:  (If none, put 0).O6c.	Decision making about whether or not to have a(nother) child.	__ __	98	Refuse to AnswerO6d.	Instruction and support for how to use methods to reduce risks of transmitting HIV infection to your partner while trying to conceive a child.	__ __	98	Refuse to AnswerO6e.	Instruction and support for use of contraception to prevent pregnancy.	__ __	98	Refuse to AnswerDid your partner attend any of these sessions/visits in the past 6 months related to:O7a.	Whether or not to have a child:	1	Yes	0	No	8	Refuse to AnswerO7b.	Use of methods to reduce transmission risks during attempts to conceive:	1	Yes	0	No	8	Refuse to AnswerO7c.	Use of contraception:	1	Yes	0	No	8	Refuse to AnswerO8.	Were any of the following barriers to your partner attending these visits? (check all that apply) f there were no barriers, partner attended all visits, select "Not Applicable".  (Check all that apply)	__	Difficult for partner to take time off from work	__	Partner doesn't think it is appropriate for him/her to attend	__	Partner is not comfortable being seen at the clinic	__	I have not told my partner about my HIV status	__	Provider did not request my partner to attend	__	My partner only wants to come when I (she) am (is) pregnant	__	It costs too much for my partner to travel to the clinic	__	Other	__	Refuse to Answer	__	Not ApplicableIf O8H is not equal to 1, then skip to O9.O8_s.	Specify other barrier(s) to your partner attending these visits:	__ __ __ __ __ __ __ __ __ __ __ __ __ __ __ __ __ __ __ __ __ __ __ __ __ __ __ __ __ __ __ __ __ __ __ __ __ __ __ __ __ __ __ __ __ __ __ __ __ __ __ __ __ __ __ __ __ __ __ __ __ __ __ __ __ __ __ __ __ __ __ __ __ __ __ __ __ __ __ __ __ __ __ __ __ __ __ __ __ __ __ __ __ __ __ __ __ __ __ __O9.	Did you decide not to attend any of these counseling sessions, or fail to attend a scheduled session, with a counselor or nurse for the purposes of family planning or safer conception counseling for any of the following reasons? (check all that apply) MOST ARE SKIPPING OUT, WHY? f all visits were attended, select "Not Applicable".  (Check all that apply)	__	My partner would not attend with me	__	My partner did not want me to attend	__	I didn't need the counseling	__	It is too inconvenient to return to the clinic for such counseling	__	It costs too much money to come to the clinic	__	I don't like the counselor/nurse	__	Other	__	Refuse to Answer	__	Not ApplicableIf O9G is not equal to 1, then skip to instruction before O10a.O9_s.	Specify other reason(s) you did not attend counseling sessions:	__ __ __ __ __ __ __ __ __ __ __ __ __ __ __ __ __ __ __ __ __ __ __ __ __ __ __ __ __ __ __ __ __ __ __ __ __ __ __ __ __ __ __ __ __ __ __ __ __ __ __ __ __ __ __ __ __ __ __ __ __ __ __ __ __ __ __ __ __ __ __ __ __ __ __ __ __ __ __ __ __ __ __ __ __ __ __ __ __ __ __ __ __ __ __ __ __ __ __ __General social supportTsai, A. C., Bangsberg, D. R., Frongillo, E. A., Hunt, P. W., Muzoora, C., Martin, J. N., & Weiser, S. D. (2012). Food insecurity, depression and the modifying role of social support among people living with HIV/AIDS in rural Uganda. Social science & medicine, 74(12).
